# Sustainable thymol-based formulations with amoxicillin and *Melaleuca alternifolia* essential oil

**DOI:** 10.3389/fchem.2026.1898133

**Published:** 2026-08-10

**Authors:** Zehra Edis, Jon Zaccary Regala

**Affiliations:** 1 Department of Pharmaceutical Sciences, College of Pharmacy and Health Sciences, Ajman University, Ajman, United Arab Emirates; 2 Center of Medical and Bio-allied Health Sciences Research, Ajman University, Ajman, United Arab Emirates; 3 Department of Biomedical Engineering, College of Engineering and Information Technology, Ajman University, Ajman, United Arab Emirates

**Keywords:** amoxicillin, antimicrobial resistance, good health and wellbeing, green synthesis, sustainability, tea tree essential oil, thymol

## Abstract

Antimicrobial resistance (AMR) against antibiotics is caused by the evolution of pathogens to endure antibiotics, rendering once powerful treatments now useless. Amoxicillin (A), a well-known antibiotic is subjected to AMR and could be more effective in combination with plant-based extracts. Tea Tree essential oil (T) and thymol (t) are known antimicrobial agents but are hampered by their lipophilicity, volatility, oxidative degradation and skin irritation. These disadvantages can be mitigated by combinations to achieve better drug delivery outcomes. Thymol, a monoterpenoid has remarkable antimicrobial activities, but several pathogens are resistant to it. T is utilized since centuries against ailments but requires nanoencapsulation due to drug-delivery problems. Formulations consisting of T, thymol, and amoxicillin (A) were investigated as possible alternatives against AMR. The best combination with highest antimicrobial properties was identified as Tt2A2, consisting of T, thymol, and A at 1:2:2 proportions, respectively. Small-sized particles of Tt2A2 were subjected to ultraviolet-visible (UV-Vis) spectrometry, Fourier transform infrared (FTIR) spectroscopy, x-ray diffraction (XRD), scanning electron microscopy (SEM), and energy dispersive x-ray (EDX) spectroscopy analysis. Tt2A2 was tested against ten microbial reference strains on discs, surgical sutures, cotton gauze bandages, surgical face masks, and KN95 masks by agar-disc diffusion methods. The formulations of Tt2A2 were effective against all ten reference pathogens achieving zones of inhibition (ZOI) of up to 30–35 mm. Antimicrobial activity of Tt2A2 is proportional to T concentrations and can be fine-tuned according to target pathogen and biomaterial. The Gram-positive *S. pneumoniae* ATCC 49619 showed high susceptibility with 33.7 mm mean ZOI and a better performance in Tt2A2^a^. The notorious Gram-negative pathogen *P. mirabilis* ATCC 29906 was inhibited with 30 mm and in average performed better in Tt2A2^b^ with higher T concentration. The formulations exhibited potential as an antimicrobial agent on surgical sutures, masks and bandages, as well as a disinfective agent. Stability studies revealed the same inhibition zones for up to 3–6 months for all 10 reference strains. However, a steady decline to intermediate inhibitory levels by Gram-positive reference strains was observed towards 18 months of storage.

## 1 Introduction

Antimicrobial resistance (AMR) is the end to the golden age of antibiotics and an introduction to a worrisome future for mankind. AMR is a result of uncontrolled misuse and overexposure of patients and other living creatures to antibiotics (Maraki et al., 2025). Nosocomial and community-acquired infections in emergency wards, hospitals, healthcare facilities and even intensive care units propel the development of multi-drug-resistant microorganisms (Diebner et al., 2025). The rise of ESKAPE pathogens (*Enterococcus faecium*, *Staphylococcus aureus*, *Klebsiella pneumoniae*, *Acinetobacter baumannii*, *Pseudomonas aeruginosa*, *Enterobacter* spp. and *Escherichia coli*) results in augmented healthcare expenditure, treatment durations, morbidity and mortality rates especially in immune-compromised patients globally (Ahmed et al., 2024; Diebner et al., 2025; Halawa et al., 2024; Maraki et al., 2025; Naghavi et al., 2024; Sakalauskiené et al., 2025). Antibiotic drugs loose steadily the grip on multi-drug resistant strains every day (Enciso-Martínez et al., 2025; Maria et al., 2025; Oliveira et al., 2024; Sodhi et al., 2021; Stohr et al., 2020). Alternatives to the existing synthetic antimicrobials are urgently needed to resolve AMR for the sake of humanity. Such alternatives could include medicinal plant extracts, essential oils, as well as combinations of these with antibiotics (Fierascu et al., 2021; Iseppi et al., 2021; Janotto et al., 2023; Lupia et al., 2024; Pezantes-Orellana et al., 2024; Romanescu et al., 2023).

The abundance of plants with a plethora of synergistic biocomponents has made them increasingly relevant in this context. Plants have been displaying commendable, well-rounded biological potential, even as antimicrobial agents for combating antimicrobial resistance (Janotto et al., 2023; Lupia et al., 2024). Naturally, they possess a synergistic array of biocomponents able to defend themselves against predators and microorganisms (Iseppi et al., 2021). Plant extracts known as essential oils (EO) contain aromatic and volatile compounds, which exhibit biological activity (Pezantes-Orellana et al., 2024). Every culture has a tradition of utilizing plant extracts and EO against ailments and illnesses since centuries before the development of synthetic antibiotics (Bertocchi et al., 2020; Ferreira et al., 2023; Gallart-Mateu et al., 2018; Ibrahium et al., 2022; Iseppi et al., 2021; Janotto et al., 2023; Johnson et al., 2022; Kairey et al., 2023; Kamel et al., 2022; Li et al., 2021; Lupia et al., 2024; Romanescu et al., 2023; Wang et al., 2021; Yasin et al., 2021; Yuan et al., 2025; Zhu et al., 2024).

Tea tree (T) oil or melaleuca oil is an EO extracted from the foliage of Australian native and endemic *Melaleuca alternifolia*, more popular under the name T. Apart from being biologically active as an anti-viral, anti-inflammatory, antioxidative, and anti-cancer agent, the antimicrobial activity of T shows promise and may be a viable solution to antimicrobial resistance (Borotová et al., 2022; Manzanelli et al., 2023; Nascimento et al., 2023). T contains a plethora of more than 38 active components with terpinene-4-ol, γ- and α-terpinene, cymene, α-terpineol, terpinolene as its major ingredients in decreasing order (Johnson et al., 2022). However, barriers for unabridged application of T include toxicity when ingested, skin irritation, allergy, irritability, volatility, lipophilicity and fast, oxidative degradation (Bertocchi et al., 2020; Kairey et al., 2023) (Table 1).

**TABLE 1:** Evidence on antimicrobial potential presented in literature.

Ingredient	Evidence on antimicrobial potential	Barriers hindering application
T	Bertocchi et al., 2020; Borotová et al., 2022; Ferreira et al., 2023; Kairey et al., 2023; Li et al., 2021; Manzanelli et al., 2023; Tisserand and Young, 2014; Wang et al., 2021; Yasin et al., 2021; Yuan et al., 2025, Yu et al., 2020	toxicity, irritation, allergy, irritability, volatility, lipophilicity, fast oxidative degradation, clinical translation
Thymol	Buriti et al., 2024; Cristea et al., 2025; Dong et al., 2024; Đorđević et al., 2025; Edis et al., 2024a; Edis et al., 2024b; Gabbai-Armelin et al., 2022; Gan et al., 2023; Garapati et al., 2023; Hajibonabi et al., 2023; Kowalczyk et al., 2020; Lopes et al., 2024; Makrygiannis et al., 2025; Malzcak and Gajda, 2023; Marchese et al., 2016; Meeran et al., 2017; Najafloo et al., 2020; Nikolic et al., 2024; Pannakal et al., 2025; Parolin et al., 2024; Raikwar et al., 2024; Ruiz et al., 2024; Sánchez García et al., 2024; Simbu et al., 2024; Vassiliou et al., 2023; Zhou et al., 2019	hydrophobicity, mutagenicity/genotoxicity (at high concentrations)
Amoxicillin	Abraha et al., 2016; Bajpai et al., 2017; Cristea et al., 2025; Đorđević et al., 2025; Gan et al., 2023; Giráldez-Pérez et al., 2023; Raikwar et al., 2024; Sharma et al., 2023; Simbu et al., 2024; Soulaimani, 2025; Taheri-Araghi, 2024	AMR, irritation, allergy, side effects

Removing such barriers includes complexing T into nano-emulsions, cellulose nanofibrils and chitosan nanoparticles (Ferreira et al., 2023; Kamel et al., 2022; Yuan et al., 2025). Furthermore, the undesired effects of T can be mitigated by preparing formulations with reduced T concentration and other essential oils or antimicrobials. Thymol, a well-known antimicrobial, is available in many essential oils and could be a suitable candidate to support T.

Thymol (t), 2-isopropyl-5-methylphenol, is a biologically-active phenolic monoterpenoid and is the dominant constituent of thyme, a historically known herb of the genus *Thymus*. Thymol is found in many other plant EO other than thyme essential oil. Thymol is considered, like T, safe (GRAS) when used at given concentrations (Đorđević et al., 2025). Furthermore, thymol has powerful antimicrobial, anti-inflammatory and antioxidant properties enabling its use in many products and biomedical applications. According to several research groups, medical applications of thymol prevent surgical site infections and bacterial proliferation on wounds (Buriti et al., 2024; Cristea et al., 2025; Ferraz, 2025; Gabbai-Armelin et al., 2022; Garapati et al., 2023; Lopes et al., 2024; Makrygiannis et al., 2025; Malzcak and Gajda, 2023; Najafloo et al., 2020; Pannakal et al., 2025; Ruiz et al., 2024; Simbu et al., 2024; Szabelski and Karpiński, 2024; Vassiliou et al., 2023). However, Gram-positive microorganisms are more susceptible to the hydrophobic thymol and T. The availability of nonpolar aromatic rings and groups compromise the thick, lipophilic peptidoglycan layer of Gram-positive pathogens causing cell permeability, membrane leakage, disrupt quorum sensing, hinder biofilm formation, and finally cell death (Gan et al., 2023; Pannakal et al., 2025; Romanescu et al., 2023). Nevertheless, thymol has like T similar disadvantages of hydrophobicity, which result in drug-delivery problems. Thymol displays at high concentrations even mutagenicity and genotoxicity (Gago et al., 2025; Sharma et al., 2023). Therefore, many investigations report thymol within formulations, nanoencapsulations and in polymeric matrices (Dong et al., 2024; Edis et al., 2024a; Edis et al., 2024b; Giotopoulou et al., 2024; Hajibonabi et al., 2023; Sánchez García et al., 2024; Sharma et al., 2023; Zamani et al., 2015; Đorđević et al., 2025). The balanced use of thymol combined with other EO, further antimicrobials and even antibiotics could abate the undesired effects of thymol (Cristea et al., 2025; Najafloo et al., 2020; Vassiliou et al., 2023).

Combinations with antibiotics appear to enhance thymol’s spectrum of antifungal, antibacterial applications (Nikolic et al., 2024). As previously studied, antibiotics like amoxicillin (A) have exhibited antimicrobial effects when used in combinations with other compounds and essential oils (Abraha et al., 2016; Bajpai et al., 2017; Giráldez-Pérez et al., 2023; Min et al., 2020; Raikwar et al., 2024; Sharma et al., 2023; Simbu et al., 2024; Soulaimani, 2025; Taheri-Araghi, 2024). Gan et al. studied gentamycin and streptomycin with thymol alone and found additive effects of these combinations against *S. aureus*, while there was no interaction with amoxicillin against *K. pneumoniae* (Gan et al., 2023). Touati et al. reviewed recently promising combinations of essential oils with antibiotics, nanoparticles, nanoemulsions and further delivery methods. They confirm the multi-target mechanisms of EO, which may be used in synergy to overcome AMR. They stress the importance of pharmacokinetics, cytotoxicity and skin-irritation related to essential oil use (Touati et al., 2025). Iseppi et al. combined EO with antibiotics and suggest using EO as adjuvants in antibiotic therapy. They stress the importance of terpinene-4-ol and 1,8-cineole on the antimicrobial and anti-biofilm activities of T. Also, they suggested reducing concentrations of each component in EO-antibiotics combinations could restore microbial sensitivity to reference antibiotics (Iseppi et al., 2023).

Depending on the antibiotic, the formulation setup and the target pathogen with its morphological characteristics, it is possible to design a potent antimicrobial and antibiofilm agent (Brożyna et al., 2021; Liu et al., 2020; Maggio et al., 2025; Touati et al., 2025). This entails that while antibiotics may not be the sole solution to combat successfully infections, their combinations with products of green chemistry becomes a captivating avenue for research. However, standardization is a limiting factor, which needs to be vehemently addressed. Essential oil production depends on environmental factors during farming, harvesting, handling, extraction methods, storage and of course type of plant material (Das and Prakash, 2024). Nevertheless, tight control on all these known factors can ensure standardized essential oils. According to Iseppi et al., combinations of the main active EO components with antibiotics could lead to promising future natural antimicrobials (Iseppi et al., 2023). Cordeiro et al. tested terpinene-4-ol in combination with different antibiotics against *S. aureus*, while Francisconi et al. used it against *Candida albicans* (Cordeiro et al., 2020; Francisconi et al., 2020). Cordeiro et al. stress the importance of combining natural EO with antibiotics against resistant strains. They suggest that the EO phytochemicals restore the sensitivity to antimicrobial drugs by reducing administration doses and related toxicity. They confirm bactericidal effects of terpinene-4-ol against *S. aureus* by compromising cell wall synthesis and biofilm formation (Cordeiro et al., 2020). Table 1 summarizes available evidence on the antimicrobial evidence of these aforementioned ingredients, as well as their limitations.

Although tea tree oil and thymol reveal inhibitory actions against microorganisms *in-vitro*, the literature lacks grounded translation into clinical practice. Reasons are insufficient proofs to support therapeutic use due to lack of professionally-designed randomized clinical trials resulting in heterogenous clinical outcomes (European Medicines Agency, 2023; Kairey et al., 2023; Yu et al., 2020). Yu et al. state in their systematic review the necessity for well-designed *in vivo* experiments, followed by comprehensive pre-clinical studies revealing preliminary information about toxicity, efficacy, safety and pharmacokinetics. The scarcity of such studies precipitate into the lack of extensive clinical trials and inability to derive clear outcomes (Yu et al., 2020). Therefore, clinical translation fails due to missing clarity regarding potency, stability, biocompatibility, bio-availability, irritation and toxicity (European Medicines Agency, 2023; Kairey et al., 2023; Yu et al., 2020). Furthermore, outcomes of those available trials are not reported adequately and the quality of the utilized tea tree oil is questionable (Kairey et al., 2023).

In this regard, adverse effects are concentration dependent and include skin irritation, as well as allergic reactions (Carson et al., 2006; Kairey et al., 2023; Scientific Committee on Consumer Safety, 2025). Such allergic reactions are a result of oxidation of tea tree oil, which triggers rapid degradation (Kairey et al., 2023). Degradation reduces α- and γ-terpinene content, but accelerates the sensitizing allergens p-cymene and peroxide (Kairey et al., 2023). Furthermore, adulteration of tea tree oil is a major problem, which increases the risks of toxicity and adverse effects (Bejar, 2017; Kairey et al., 2023). According to Tisserand et al., tea tree oil is considered safe with concentrations below 15% for topical use, while the further applications through the respiratory tract are not recommended (Kairey et al., 2023; Tisserand and Young, 2014). Tea tree oil is not recommended for children below 12 years old and patients with hypersensitivity (European Medicines Agency, 2023. Tea tree oil is considered safe for four cosmetic product categories at specified maximum concentrations between 2% and 0.1% (Scientific Committee on Consumer Safety, 2025).

This study aims to assess antimicrobial activity of thymol, amoxicillin (A), and T in combined form. The existing literature does not contain peer-reviewed studies for the combination of tea tree oil-thymol-amoxicillin. Accordingly, this classifies our investigation as a novel experimental formulation filling the knowledge gap. However, there are few studies describing combinations of essential oils with thymol and other antibiotics, including several β-lactam antibiotics (Bassolé and Juliani, 2012; Langeveld et al., 2014; Nazzaro et al., 2013).

Based on our previous works, different samples and formulations were investigated extensively to identify the best antimicrobial combinations in this study. These included T, t, A, terpinene-4-ol (T-4-ol), their mixtures Tt, TtA, Tt1.5, Tt1.5A, Tt1.5A2, Tt2, Tt2A and Tt2A2, in which the numbers specify the proportion of used ingredient. The trials with T2 were not mentioned in this paper, because increased essential oil in the formulations had an inverse effect on antimicrobial activities due to reduced solubilities in water and other polar solvents. In this study, only those relevant to the title compound were mentioned in the antimicrobial testing results. The investigation identified the formulation Tt2A2 with the highest inhibitory effects against the 10 reference strains with the proportions 1:2:2 of T, t, and A, respectively. These resulted in homogenous, small-sized particles without the use of nanotechnology and its required additives (Supplementary Material S5). Spectrophotometric analysis, microscopic visualization and assessment of antimicrobial activity on biomedical tools were utilized to describe the results. However, no study evaluated concentration dependency in formulations with thymol, amoxicillin and T. Accordingly, the antimicrobial activity of Tt2A2 is also dependent on the thymol concentration and acts differently on the studied pathogens in each case. In this study, the formulations were narrowed down to Tt2A2^a^ and Tt2A2^b^, which exhibited the highest antimicrobial properties in comparison to other combinations. Tt2A2^b^, the thymol-rich combination with double the concentration of the former has distinct activities against several pathogens from the selected strains, while its antifungal activity plummeted. The two formulations Tt2A2^a^ and Tt2A2^b^ and their practical applications against 10 microbial reference strains on sterile cotton bandages, surgical polyglycolic acid (PGA) sutures, surgical face masks, and KN95 face masks were explored (Edis et al., 2024; Edis and Bloukh, 2024). In this regard, impregnating biomaterials with Tt2A2 changes them from passive to bioactive state within the framework of sustainability. Biofunctionalization of these homogenous materials could be of advantage especially due to their cost-effectivity, simple preparation and availability (Ferraz, 2025). These outcomes indicate increased antimicrobial activity to reduce inflammatory processes by mitigating microbial proliferation, and increased protection against respiratory tract infection (Hajibonabi et al., 2023). There are interdependencies on utilized thymol concentration, coated material and cell membrane morphology of the studied 10 reference pathogens. Nevertheless, further *in-vivo* studies are warranted to investigate the full extent of biofunctionalization of the title formulation Tt2A2.

## 2 Materials and methods

### 2.1 Materials

Amoxicillin was purchased from Sigma-Aldrich Chemical Co. (Shanghai, China). T, thymol, terpinene-4-ol, the solvent ethanol absolute, Mueller Hinton broth, Sabouraud Dextrose broth, and the reference microbe strains *Bacillus subtilis* WDCM 0003 Vitroids, *E. coli* WDCM 00013 Vitroids, *P. aeruginosa* WDCM 00026 Vitroids, *K. pneumoniae* WDCM 00097 Vitroids, and *C. albicans* WDCM 00054 Vitroids were purchased from Sigma-Aldrich Chemical Co. (Missouri, the United States of America). Another solvent was ultrapure distilled water.

The strains *Streptococcus pyogenes* ATCC 19615, *S. aureus* ATCC 25923, *Enterococcus faecalis* ATCC 29212, *Proteus mirabilis* ATCC 29906, and *Streptococcus pneumoniae* ATCC 49619, McFarland standard sets, disposable sterilized Petri dishes containing Mueller Hinton II agar, and antibiotic discs of gentamicin (9,125, 30 µg/disc) and nystatin (9,078, 100 IU/disc) were obtained from Liofilchem (Roseto degli Abruzzi, Italy).

Sterile filter paper discs of 6 mm (mm) diameter were supplied by Himedia (Maharashtra, India). Sterile surgical sutures from polyglycolic acid of quality USP: 3-0, Metric: 2, 19 mm, 75 cm, DC3K19 were obtained from General Medical Disposable (Istanbul, Turkey). KN95 face masks (Hubei Province, China), disposable surgical 2-ply non-woven face masks, and sterile cotton gauze bandages were purchased locally from a pharmacy. All reagents were of analytical grade, and sterile conditions were ensured for use.

### 2.2 Preparation of sample Tt2A2

The samples were prepared by of first dissolving 0.15 g of T and 0.155 g of thymol in 10 mL pure ethanol each in two separate beakers at room temperature (RT) and constant stirring. Meanwhile, 0.025 g amoxicillin (A) was completely dissolved in 50 mL of distilled water by constant stirring at RT for 30 min. Afterwards, Tt2A2 was prepared through a one-pot method at room temperature under constant stirring by adding first one part T dissolved in ethanol, then two parts thymol dissolved in ethanol, and finally two parts amoxicillin dissolved in water according to 1:2:2, respectively. However, the antimicrobial results dependency on the thymol concentration culminated into refining the formulations as Tt2A2^a^ and Tt2A2^b^. In comparison, Tt2A2^a^ contains 0.155 g of T oil in 10 mL of pure ethanol, 0.15 g of thymol in 10 mL of pure ethanol, 0.025 g of amoxicillin completely dissolved in 50 mL of distilled water with amoxicillin concentrations of 1.368 μg/mL and 0.068 mmol Tt2A2^b^ consists of 0.155 g of T in 10 mL of pure ethanol, 0.15 g of thymol in 5 mL of pure ethanol, 0.025 g of amoxicillin in 50 mL of distilled water as above. All the formulations are clear liquids with pH 5. The samples were kept tightly sealed in the fridge at 3 °C for further use.

### 2.3 Characterization and analysis of sample Tt2A2

#### 2.3.1 Scanning electron microscopy (SEM) and energy-dispersive X-ray spectroscopy (EDS)

Sample Tt2A2 underwent analysis on the VEGA3 supplied by TESCAN (Brno, Czech Republic) at 15 kV. One drop of sample Tt2A2 was extracted and diluted with distilled water, before being set on the carbon-coated copper grid and dried. Above sample was coated with gold by the Mini Sputter Coater supplied by Quorum Technologies (Brno, Czech Republic). Morphology was determined by SEM, and purity of sample Tt2A2 was verified by EDS (Akhtar et al., 2024; Najjar and Bridge, 2020).

#### 2.3.2 Ultraviolet-visible (UV-Vis) spectrometry

The title formulation Tt2A2 was tested on the 2600i UV-Vis Spectrophotometer supplied by Shimadzu (Kyoto, Japan) in the wavelength range from 195 to 800 nm (nm) (Rawat et al., 2017). Testing was done on pure sample Tt2A2, then 10, 25, 50, and 70-time dilutions using ethanol absolute.

#### 2.3.3 Fourier-transform infrared (FTIR) spectroscopy

The title formulation Tt2A2 underwent analysis on the ATR IR spectrometer with a Diamond window supplied by Shimadzu (Kyoto, Japan) in the wavelength range from 400 to 4,000 inverse centimeters (cm^-1^) (Pinto et al., 2024).

#### 2.3.4 X-ray diffraction (XRD)

The title formulation Tt2A2 was analyzed on the X-ray Diffractometer supplied by BRUKER (Karlsruhe, Germany) using copper radiation of wavelength 1.54060, coupled Two Theta/Theta, time/step of 0.5 s and step size of 0.03 (Pinto et al., 2024).

### 2.4 Determination of antimicrobial activity

The title formulation Tt2A2 underwent testing of antimicrobial activity against the ten aforementioned reference strains, comprised of nine bacterial strains and one fungus. The Kirby-Bauer methods were applied (Soulaimani, 2025). Positive control was employed, with reference antibiotics nystatin and gentamicin. Biomedical tools bandages, sutures, surgical face masks, and KN95 face masks were impregnated separately with 2 mL of Tt2A2, kept at ambient temperature for 2 h and assessed consequently to standalone sample assessment.

#### 2.4.1 Bacterial strains and culturing

Testing of antimicrobial activity was prepared by storing the reference strains of concentrations 1 × 10^6^ CFU per milliliter at −20 °C. Inoculation of fresh microbes to Mueller Hinton broth followed, and inoculations were stored at 4 °C for storage and future use.

#### 2.4.2 Zone of inhibition (ZOI) plate preparation

Bacterial reference strains were suspended in 10 mL of Mueller Hinton broth. Following this was incubation from two to 4 hours at 37 °C (Soulaimani, 2025). Fungal reference strain C. albicans WDCM 00054 was similarly suspended in Sabouraud Dextrose broth and incubated at 30 °C.

The resulting cultures were adjusted to the 0.5 McFarland standard. To coat the Petri dishes, sterile cotton swabs of 100 μL of the microbial cultures were used. Plates were dried for 10 minutes before antimicrobial testing.

#### 2.4.3 Disc diffusion

The disc diffusion method for antimicrobial testing was employed according to the Clinical and Laboratory Standards Institute’s (CLSI) recommendations (Touati et al., 2025). Additionally, sterile filter paper discs, cotton bandages, surgical sutures, surgical face masks, and KN95 face masks were doused in 2 mL each of the samples, duration of 24 h. Antimicrobial testing was executed by placing dip-coated biomedical tools in prepared microbials. The diameter of the clear area, called zone of inhibition (ZOI) was measured to the nearest millimeters by using standard rulers. Antimicrobial activity was observed if clear, enunciated ZOIs were observed. Absence of any zone of inhibition (ZOI = 0 mm) around the samples equaled resistance (R) against the utilized reference. All antimicrobial tests were repeated by three fresh biological replicates at different days in standard Himedia agar plates to ensure almost similar results under controlled conditions. Negative controls were resistant and were not mentioned further. Gentamycin and nystatin were used as positive controls.

### 2.5 Statistical analysis

Statistical analysis was performed by SPSS software (IBM SPSS Statistics, Version 30.0.0.0 (172), 2024, SPSS Inc., Chicago, Il, USA) with data presented in mean values. The significance between groups was determined through one-way ANOVA, followed by Tukey’s *post hoc* analysis. Statistical significance was defined as p < 0.05. Data are presented as mean ± standard deviation (SD) from three replicate measurements. These produced for each of the three groups df = 2 and df = 6 in the ANOVA. Prior to one-way ANOVA, normality was assessed using the Shapiro–Wilk test and homogeneity of variances was evaluated using Levene’s test. All ANOVA statistics are based on triplicate agar diffusion assays for each microbial strain and formulation.

## 3 Results and discussion

### 3.1 Characterization and analysis of formulation Tt2A2

The title formulation Tt2A2 underwent analytical characterization to investigate its morphological characteristics, composition and antimicrobial properties.

#### 3.1.1 Scanning electron microscopy (SEM) and energy-dispersive X-ray spectroscopy (EDS)

**FIGURE 1:**
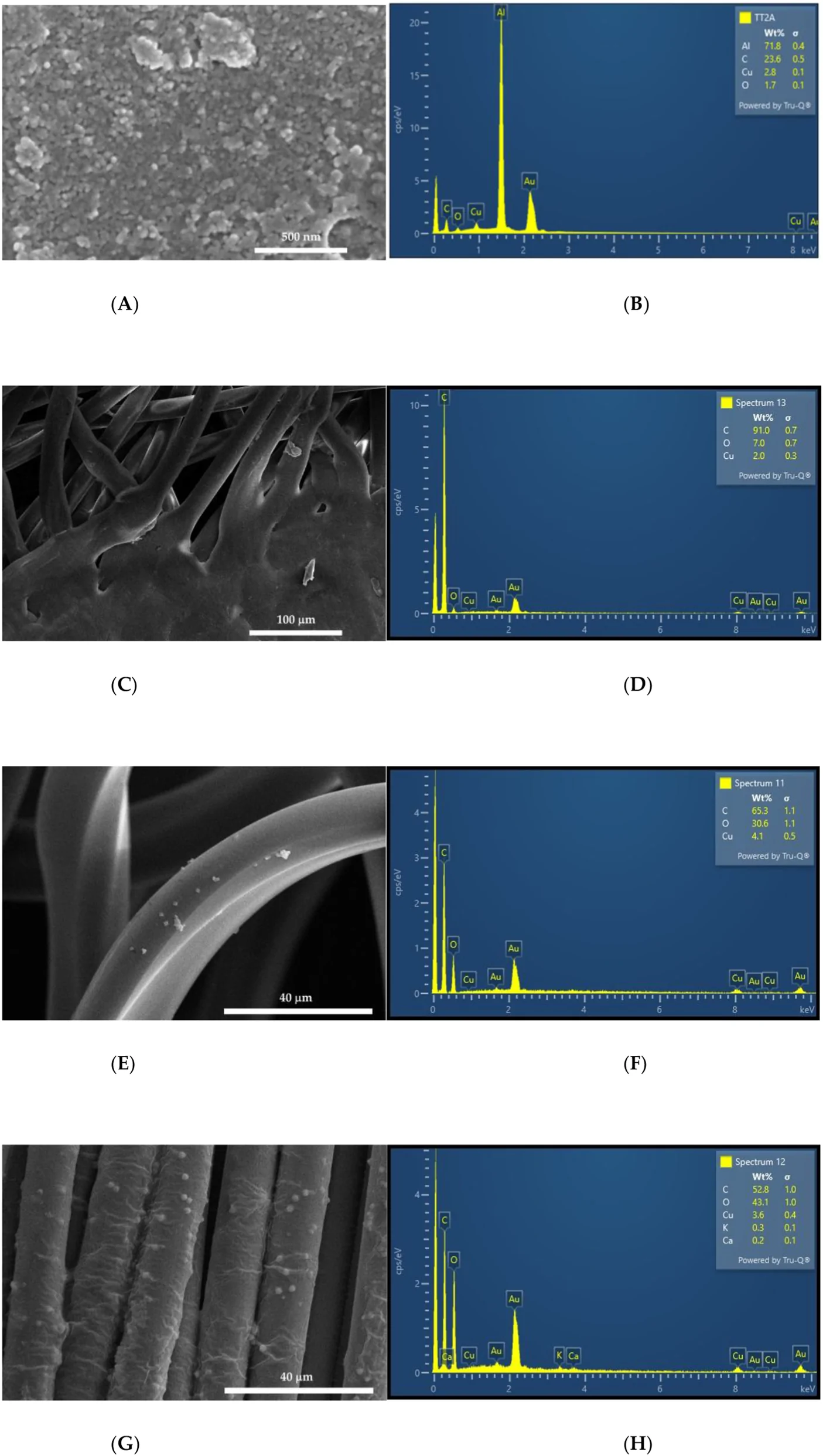
SEM and EDS analyses. Sample Tt2A2, as standalone sample **(A,B)**, in impregnated face masks **(C,D)**, in impregnated cotton gauze bandages **(E,F)**, and in impregnated surgical sutures **(G,H)**. The magnification values are 100,000 x at 10.00 kV (Figure 1A), 500 x at 20 kV (Figure 1C), 2000 x at 20 kV (Figures 1E,G).

SEM and EDS analysis were performed on the title formulation Tt2A2 to investigate its morphology and composition (Figure 1).

In Figure 1A, formulation Tt2A2 appears small-sized, spherical and homogenous from SEM, with cohesive convolutions in several areas (S5). Identified in the sample through EDS are carbon (23.6%), copper (2.8%), and oxygen (1.7%), reflected in Figure 1B. The observed elements can be sourced from T, thymol, and amoxicillin, which constitute sample Tt2A2. The EDS confirms the purity of the compound. Gold and aluminum have been observed in the EDS samples due to the material of the device and the applied coating of the sample.

Furthermore, SEM and EDS analysis was conducted on masks, bandages, and sutures impregnated with Tt2A2. Figures 1C,D show the SEM and EDS of face masks impregnated with sample Tt2A2, respectively. On masks, SEM appears homogeneously distributed, showing a relatively even coating. Carbon (91%), oxygen (7%), and copper (2%) were identified, as found in the EDS result of standalone sample Tt2A2. Carbon detection increased significantly in comparison, with changes also observed for oxygen and copper concentrations. These changes may be a result of the analysis of the face mask material.

Figures 1E,F show the SEM and EDS of bandages impregnated with sample Tt2A2, respectively. While generally homogeneous as in aforementioned results, individual bits and clumps can be identified in specific areas. This imaging was done at the 40 nm scale. Again, carbon (65.3%), oxygen (30.6%), and copper (4.1%) were detected and identified. Changes may again be hypothesized to be a result of difference in material, from standalone sample to face masks to bandages.

On the surgical sutures, exquisite spheres of formulation Tt2A2 can be observed through the results of SEM in Figure 1G. Apart from the recurring identifications of carbon (52.8%), oxygen (43.1%), and copper (3.6%), traces of potassium (0.3%) and calcium (0.2%) were detected and identified, and can be seen in Figure 1H.

#### 3.1.2 Ultraviolet-visible (UV-Vis) spectrometry

**FIGURE 2:**
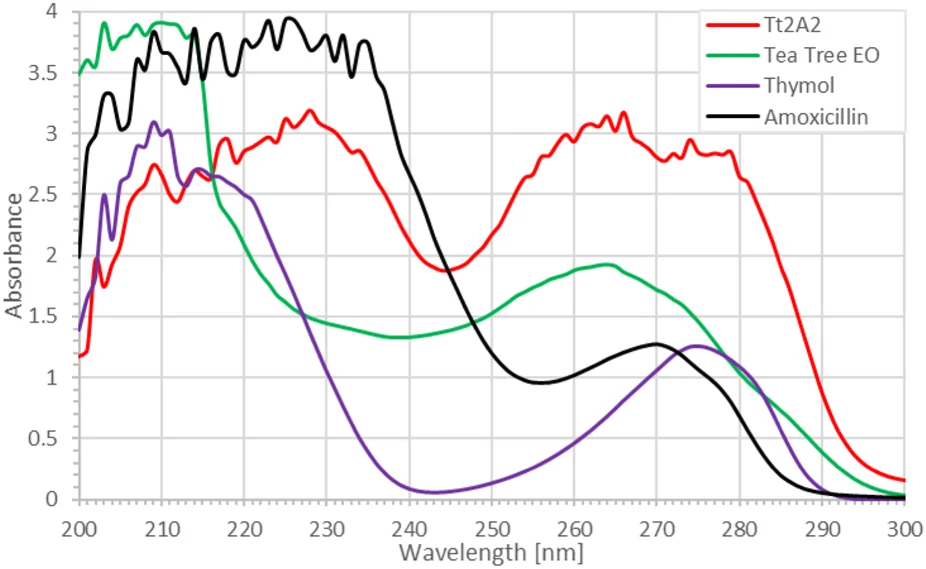
UV-vis spectra of Tt2A2, T, thymol, and amoxicillin.

The UV-Vis spectra of the title formulation Tt2A2 and its ingredients is shown in Figure 2.

Table 2 is a comparison of the UV-Vis spectra of sample Tt2A2 and its ingredients, alongside results from references to confirm purity.

**TABLE 2:** UV-Vis spectra of Tt2A2, T, thymol, and amoxicillin in comparison to previous investigations (Abraha et al., 2016; Edis et al., 2024a; Ibrahium et al., 2022).

Sample Tt2A2	T	T (Ibrahium et al., 2022)	Thymol	Thymol (Edis et al., 2024a)	Amoxicillin	Amoxicillin (Abraha et al., 2016)
​	201 vs.	201 s	​	​	​	​
202 m	203 vs.	​	203 s	203 s	​	​
204 m,sh	​	​	​	​	204 vs.	​
​	205 vs., sh	​	205 s, sh	205 s	​	​
206 s, sh	207 vs.	​	207 s	207 s	207 vs.	​
209 s	209 vs., sh	​	209 vs.	209 vs.	209 vs.	​
​	210 vs., br	​	​	210 vs.	​	​
211 s, sh	211 vs., sh	​	211 vs.	​	211 vs., sh	211 w
214 s, s	214 vs.	​	214 s, br	​	214 vs.	​
​	​	​	215 s	216 s	216 vs., br	​
217 s, br	​	​	217 s, sh, br	​	​	​
218 s, sh, br	218 s, sh	​	219 s, sh, br	​	​	​
220 s, sh	​	​	221 s, sh, br	220 s	220 s	​
​	222 m	222 vs.	​	​	​	​
223 s	223 m, sh	223 vs.	​	​	223 vs.	​
225 s	225 m, sh	225 vs.	​	​	225 vs., br	​
228 vs.	227 m, sh	​	​	​	​	​
229 vs., sh	​	​	​	​	​	​
​	​	​	​	​	230 vs.	230 m
231 s, sh	​	​	​	​	231 vs.	​
234 s, sh	​	​	​	​	234 vs., br	​
237 s, sh	​	​	​	​	237 vs., sh	​
​	​	242 vs.	​	​	241 s, sh, br	​
253 s, sh	253 m, sh	253 vs.	​	​	​	​
255 s	255 m	255 vs.	​	​	​	​
256 s, sh	256 m, sh	​	​	​	​	​
​	258 m	258 vs.	​	​	​	​
259 s	​	260 vs.	​	​	​	​
262 s	262 m, sh	​	​	​	​	​
263 s	263 m, br	​	​	​	​	​
264 s	264 m, br	​	​	​	​	​
​	​	265 vs.	​	​	​	​
266 s	266 m, br	​	​	​	​	​
269 s, sh, br	269 m, sh, br	269 vs.	​	​	​	270 w
272 s	272 m, sh, br	​	​	​	272 m	​
273 s	​	​	​	​	​	​
274 s	274 m, sh, br	​	​	​	​	​
275 s	275 m, sh, br	​	275 m, br	​	​	​
278 s 279 s	​	​	278 m, sh, br	277 m	278 w, sh, br	​
281 s, sh	​	​	282 w, sh	​	​	​
285 m, sh, br	285 w, sh, br	​	​	​	​	​

vw = very weak, w = weak, m = moderate, s = strong, vs. = very strong, br = broad, sh = shoulder.

Through corresponding peaks across each spectrum, sample Tt2A2 appears to contain molecules from each ingredient. While amoxicillin, T and t exhibit very intense peaks in the range 200–245 nm, the manifestations in sample Tt2A2 are of strong nature, which could dictate antagonism. The high intensity absorption can also be attributed to the same strong description of the peaks of thymol before 220 nm as experienced in previous investigations (Edis et al., 2024a; Edis et al., 2024b). From around 250 nm–300 nm, the mixture of each ingredient is again especially indicated, reflected both quantitatively and qualitatively with a potential favor toward the constituents of T and t. In this region, the absorption of T, t and A are all overlapping and are represented in the graph of the formulation Tt2A2 with a higher absorption intensity, which could be due to an additive effect. The increase of the absorption intensity of the formulation in this wavelength region is related to an increase of chromophores, decrease of hydrogen bonding and encapsulation of the involved molecules (Edis et al., 2024a; Edis et al., 2024b). However, a more detailed description is needed due to the diversity of components within T.

The absorption peaks at 205, 266 and 225 nm in T are according to Johnson et al., indicative of terpinene-4-ol, α- and γ-terpinene, respectively (Johnson et al., 2022). Terpinene-4-ol is a major ingredient in T and appears with high absorption intensities in the UV-vis analysis at 203, 205 and 212 nm (Figure 2; Table 2) (Johnson et al., 2022). After adding thymol and A into T, terpinene-4-ol (T4) absorption peak intensities are drastically decreased and undergo blue shifts towards 202, 204 and 209 nm, respectively (Figure 2; Table 2). These results are advocating the engagement of terpinene-4-ol into more hydrogen bonding, less availability of chromophores, saturation of its C=C bond to C-C and increase in encapsulation within the formulation Tt2A2. These findings could confirm, that the π bond of terpinene-4-ol is removed by reduction within Tt2A2.

Further minor components of T are γ-terpinene and α-terpinene (Johnson et al., 2022). The former absorbs at 219, 223 and 225 nm and the latter at 266 nm (Figure 2; Table 2) (Ibrahium et al., 2022; Johnson et al., 2022). Both components display increased intensity peaks within Tt2A2 like α-terpineol and p-cymene, which are more minor components in T (Johnson et al., 2022). Johnson et al. report α-terpineol absorption at 281 nm (Johnson et al., 2022). In the title formulation, α-terpineol peaks are at 275 and 285 nm and increase in absorption intensity within the formulation Tt2A2 (Figure 2) (Johnson et al., 2022). The observed hyperchromic effect indicates increased availability of conjugate systems, or chromophores in α-terpineol once it is mixed into the formulation. The α-terpineol in the Tt2A2 seems to be less encapsulated than the free α-terpineol in T, which was dissolved in ethanol. In Tt2A2, the α-terpineol seems to engage into less hydrogen bonding. The terpene p-cymene appears as a medium sized shoulder in T but increases absorption intensity in Tt2A2 (Figure 2; Table 2). The related hyperchromic effect is related to higher concentration of p-cymene, which is formed by oxidation in Tt2A2.

Thymol shows several absorption peaks in the UV-vis spectrum in accordance to our previous studies (Figure 2; Table 2) (Edis et al., 2024a; Edis et al., 2024b). After mixing into the formulation Tt2A2, most of the absorption peaks are the same, except in two cases with red shifts. The first bathochromic effect is noticed by a shift from 221 nm to 234 nm in pure thymol (t) compared to Tt2A2, respectively (Figure 2). The other red shift appears at 282 nm towards 285 nm and is accompanied by an increase in absorption intensity, as well (Figure 2). The latter finding confirms, that the aromatic π bond system of thymol is restored after preparing the formulation Tt2A2. This suggestion is ratified by thymol experiencing a bathochromic shift in the 210–240 nm range, alongside an increase in absorption intensity. Consequently, this finding indicates an increase in size by less complexation and/or reinstating the aromatic conjugation system of the thymol molecule within Tt2A2 (Edis et al., 2024a).

Amoxicillin governs the UV-vis spectrum between the wavelengths 200–255 nm due to its rich molecular structure and many functional groups (Abraha et al., 2016). In the 200–240 nm range, a decrease in the intensity of amoxicillin’s peaks can be significantly observed in comparison to Tt2A2. Furthermore, one blue shift in combination with a hypochromic effect is observed, when pure A moves into the formulation Tt2A2 from 241 nm to 237 nm, respectively (Figure 2). However, this narrative changes in the 255–290 nm range, wherein increase in intensity is inferred when moving from pure A to Tt2A2 (Abraha et al., 2016). This suggests, that A is in Tt2A2 less encapsulated and engages in less hydrogen bonding. Similar to thymol’s transformation, an increase in conjugation systems occurs from C-C to C=C or extension of π systems.

#### 3.1.3 Fourier-transform infrared (FTIR) spectroscopy

**FIGURE 3:**
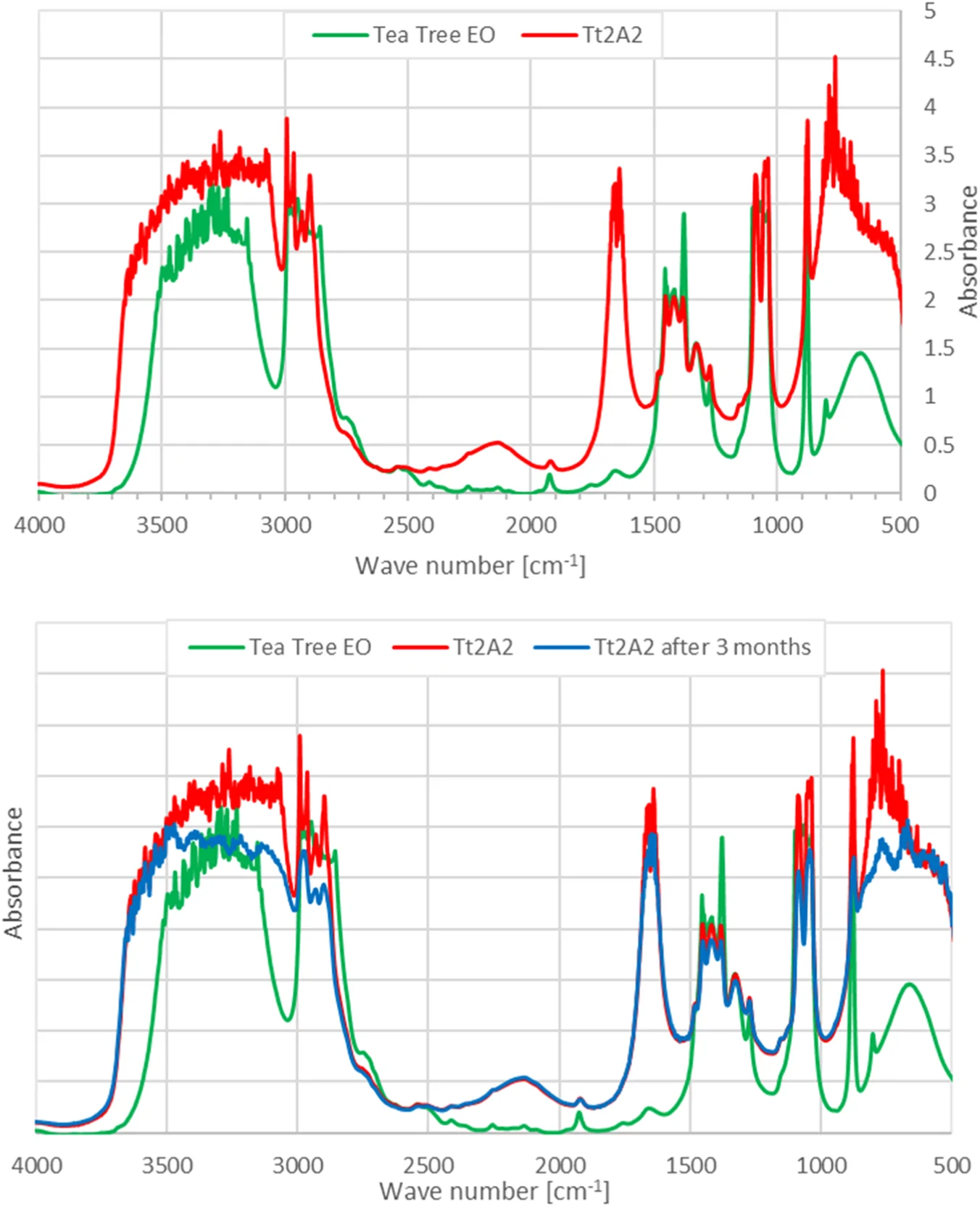
FTIR spectrum of sample T (green), Tt2A2 (red) and below with Tt2A2 after 3 months (blue).

The FTIR spectrum of formulation Tt2A2 can be seen in Figure 3 in comparison to T.

Figure 3 shows the difference between pure T (green graph) and the formulation Tt2A2 (red graph). The wavenumber region between 3,600 to 3,000 cm^-1^ is related to carboxyl- and hydroxyl groups, as well as water in the samples (Edis et al., 2024a; Edis et al., 2024b; Gallart-Mateu et al., 2018; Min et al., 2020; Sharma et al., 2023). The green curve is T in ethanolic solution. After preparing the formulation Tt2A2 (red curve), broadening is observed in the absorption spectrum due hydroxyl groups in thymol (t), carboxyl group in A and the availability of water as a solvent for amoxicillin A. Consequently, the broadening confirms a formation of a widened hydrogen bonding network within the formulation. The bands in the region from 2,990 to 2,857 cm^-1^ in Figure 3 correspond to the CH3- and CH2- vibrational modes originating from thymol, A, and T ingredients terpinene-4-ol (T4), γ-terpinene and α-terpinene, as well as α-terpineol and terpinolene (Edis et al., 2024a; Edis et al., 2024b; Ferreira et al., 2023; Gallart-Mateu et al., 2018; Min et al., 2020; Sharma et al., 2023; Zamani et al., 2015). However, the red curve of TT2A2 displays a slight blue shift with increased absorption intensities in comparison to pure T (green curve) (Figure 3). The hypsochromic effect towards higher frequencies and therefore higher energies is combined with a slight increase in absorbance. Consequently, this finding indicates the increased availability of more methyl- and methylene groups interacting with the IR light. Therefore, these groups absorb intensely due to their higher concentration in Tt2A2. This confirms stronger bond formation within the carbon-carbon units, which are less complexed and increasingly free in contrast to the other polar groups, which are highly affected by hydrogen bonding. The absorption bands in the region from 2,510 to 1,153 cm^-1^ are related to C-O- and C=C aromatic vibrational stretching, while those from 1,101 to 1,037 cm^-1^ correspond to C-O and C-N stretching vibrations (Figure 3; Table 3) (Ferreira et al., 2023; Gallart-Mateu et al., 2018; Giotopoulou et al., 2024; Min et al., 2020; Zamani et al., 2015).

**TABLE 3:** FTIR analysis of sample Tt2A2 in comparison to ingredients (Gallart-Mateu et al., 2018; Giotopoulou et al., 2024; Min et al., 2020; Sharma et al., 2023; Zamani et al., 2015).

​	ν_1,2_ (O-H)_s,a_ ν (COOH)_a_	ν (C-H)_a_	ν (C-H)s	ν(C=C)_a_ ν(C=O)_a_	δ (C-H)_a_ δ (CH_2_) δ (O-H)	ν(C=C)_s_ ν(C-C)_s_	ν(C-O)	ν(C-O) ν(C-N)
Tt2A2	3,582 s 3,541 s 3,469 s	2,992 s 2,986 vs.,sh 2,963 s	2,899 vs. 2,891 vs.,sh	1,666 m 1,637 m 1,641 m	1,487 m,sh 1,454 m 1,420 m	1,385 m 1,327 m	1,275 s	1,090 vs. 1,055 vs. 1,051 vs.
	3,451 vs. 3,412 vs. 3,286 vs. 3,262 vs. 3,068 s	2,930 m 2,902 vs.,sh	​	​	1,418 vs. 884 vs. 878 vs. 802 vs. 821 s 813 s 802 s 791 s 766 vs. 756 vs. 731 vs. 704 vs. 692 vs. 677 s 635 s	​	​	1,038 vs.
T	3,468 s 3,458 s 3,435 s 3,279 s 3,154 s	2,990 s 2,967 s 2,947 s 2,924 s 2,912 s	2,889 s 2,857 s	1757 w 1,659 vw	1,510 w,sh 1,485 m,sh 1,457 vs. 1,423 sh 1,418 vs. 1,379 vs. 930 w,sh	1,379 vs. 1,329 m	1,275 m 1,153 w,sh	1,101 vs. 1,072 sh 1,065 vs. 1,062 vs. 1,037 vs.
​	​	​	​	​	885 vs. 878 vs. 802 m 665 s,br	​	​	​
T (Gallart-Mateu et al., 2018)	3,450 m	2,961 s	2,921 s	1,650 vw	1,468 s	1,300 m	1,250 m	1,085 m
	​	​	2,876 s	​	1,444 s	​	​	1,032 m
	​	​	​	​	1,378 s	​	​	​
Thymol (Zamani et al., 2015)	3,219 s	2,961 s	2,936 s	1,620 w	1,458 m	1,320 w	1,268 m	1,059 m
	​	​	2,868 m	1,583 w	1,423 s	​	1,246 w	1,033 vw
	​	​	​	1,524 w	​	​	​	​
Thymol (Giotopoulou et al., 2024)	​	​	​	1,618 w 1,585 w	1,457 m 1,419 m	1,379 m 1,362 m 1,343 m	1,228 m	1,152 m 1,088 s 945 s 806 s
Thymol (Sharma et al., 2023)	3,201 s 3,169 s 3,103 s 3,066 s 3,036 s	​	2,929 s	1,622 w 1,585 s	1,458 vs.	​	​	1,091 s 1,058 s
Amoxicillin (Min et al., 2020)	3,530 w 3,425 w 3,150 m	2,960 m	​	1750 vs. 1,680 vs. 1,620 m	1,490 m 1,470 vs. 1,420 s	1,375 s	1,225 vs.	1,090 m
​	3,025 m	​	​	1,590 vs.	910 w	​	​	​
​	​	​	​	1,510 s	840 w	​	​	​

vw = very weak; w = weak; m = medium; s = strong; vs. = very strong; br = broad; δ = bending; ν = vibrational stretching; s = symmetrical; a = asymmetrical, sh = shoulder.

In general, Tt2A2 displays increased absorption intensity in all wavelength regions, except for the absorption bands at around 1,385, 1,418, 1,420 and 1,454 cm^-1^ (Figure 3; Table 3). The hyperchromic shifts in almost all the FTIR spectrum are indicative of the increase of the concentration of polar functional groups by adding the three solutions of T, thymol (t) and A into the formulations. Their polar functional groups, as well as the solvents ethanol and water interact with light. The increase of absorption goes along with more groups interacting with the light from thymol, terpinene-4-ol (T4), as well as the carboxylic acid- and ketone groups in A. Furthermore, the only peaks of the FTIR spectrum, where a decrease of absorption intensities happen after preparing the formulation Tt2A2 are 1,385, 1,418, 1,420 and 1,454 cm^-1^ (Figure 3; Table 3). Consequently, the related functional groups, which were present in T (green curve) showed higher absorption intensities because of their ability to interact with the light. These absorption bands are related to the major constituent of T, terpinene-4-ol at 1,379 cm^-1^, as well as γ-terpinene and α-terpinene at 1,457 and 1,418 cm^-1^, respectively (Figure 3; Table 3) (Ferreira et al., 2023). After adding the other components thymol (t) and A into T to prepare the formulation Tt2A2, these bands manifest themselves at lower absorption intensities the red curve (Figure 3; Table 3). Within the formulation Tt2A2, the aromatic C=C groups in terpinene-4-ol (1,379 cm^-1^), γ-terpinene (1,418 cm^-1^) and α-terpinene (1,454 cm^-1^) have reduced ability to interact with the light because they are encapsulated and their chromophores cannot absorb (Ferreira et al., 2023). Meanwhile, the C-O groups within the hydroxyl group in terpinene-4-ol, thymol and A display low intensity absorptions around 1,420 cm^-1^ due to increased hydrogen bonding (Figure 3; Table 3) (Edis et al., 2024a; Edis et al., 2024b; Gallart-Mateu et al., 2018; Giotopoulou et al., 2024; Min et al., 2020; Zamani et al., 2015). These findings underscore the results of the UV-vis analysis (Figure 2; Table 2).

The title formulation Tt2A2 displays an increased absorption band at 1,666 cm^-1^ related to C=O functional groups within A (Figure 3; Table 3) (Min et al., 2020). The other absorption bands at 1,641 and 1,637 cm^-1^ originate from the C=C bonds within thymol and terpinolene, respectively (Figure 3; Table 3) (Edis et al., 2024a; Edis et al., 2024b; Giotopoulou et al., 2024; Min et al., 2020; Zamani et al., 2015). The complexity of the FTIR spectrum could be attributed to the interactions between thymol and the dominant alcohols (terpinene-4-ol) and alkenes (α- and γ-terpinene) of T, as well as with the penicillin nature of amoxicillin (Edis et al., 2024a; Edis et al., 2024b; Ferreira et al., 2023; Gallart-Mateu et al., 2018; Giotopoulou et al., 2024; Min et al., 2020; Zamani et al., 2015).

The FTIR spectrum of Tt2A2 after storage of 3 months confirms the stability of the title compound (Figure 3, blue curve). The formulation was stored in darkness in the fridge. The overall FTIR spectrum indicates strongly almost in every band region a hypsochromic effect. The decrease of intensity for all functional groups affirms encapsulation with less availability of chromophores, and more hydrogen bonding within the sample. The region from 2,510 to 1,153 cm^-1^ is an exception. The related to C-O- and C=C aromatic vibrational stretching bands do not reduce the intensity after a duration of 3 months. These functional groups do not engage in complexation during 3 months of storage and remain therefore free of hydrogen bonding or complexation.

#### 3.1.4 X-ray diffraction (XRD)

**FIGURE 4:**
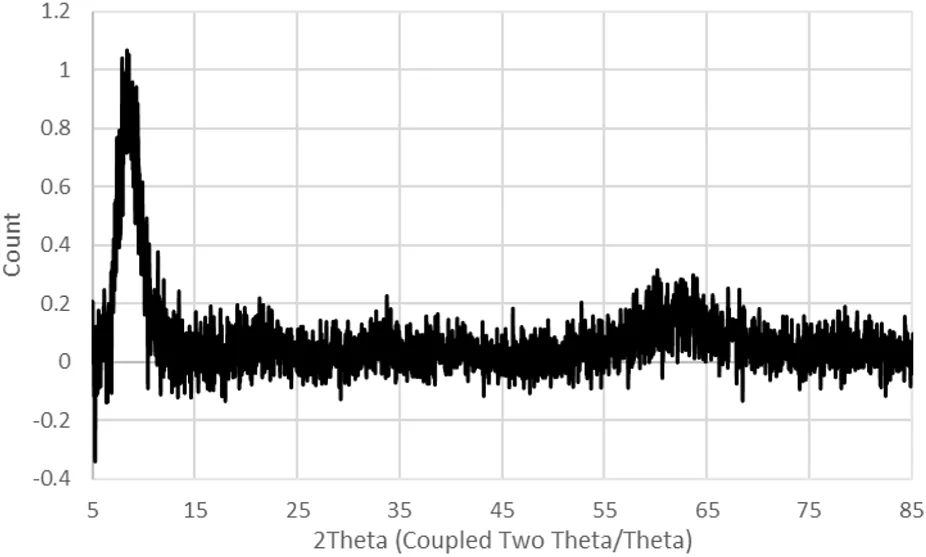
X-ray diffraction (XRD) analysis of Tt2A2.

The XRD spectrum of sample Tt2A2 is seen in Figure 4, and the analysis is outlined in Table 4.

**TABLE 4:** XRD analysis of the formulation constituents T, thymol, and amoxicillin, in comparison to the sample TT2A2 itself.

Group	T (Zhu et al., 2024)	Thymol (t) (Edis et al., 2024)	Thymol (t) (Sharma et al., 2023)	Amoxicillin (A) (Bajpai et al., 2017)	Tt2A2
T	10.36 m	​	​	​	9.65 vs.
	18.18 m	​	​	​	10.28 m
	43.68 vw	​	​	​	46.06 vw
Thymol	​	​	7.0 vs. 8.0 w 11.78 w 11.95 vw 15.77 w 16.62 vs.	​	7.7 m 8.38 vs. 11.40 w 11.97 w
	​	24.42 vw	18.71 vs.	​	​
	​	28.83 vw	20.3 m	​	​
	​	30.08 m 30.14 w	20.76 m 25.35 s	​	21.69 m
Amoxicillin	​	​	​	12.25 w	13.45 w
	​	​	​	15.50 vs.	​
	​	​	​	19.00 vs.	​
	​	​	​	20.25 m	21.00 w
	​	​	​	24.20 m	​
	​	​	​	26.50 w	​
	​	​	​	29.00 m	​
	​	​	​	32.25 vw	33.76 w
	​	​	​	35.00 vw	​
	​	​	​	40.00 w	63.00 w,br

* vw = very weak; w = weak; m = moderate; s = strong; vs. = very strong; br = broad.

The title formulation Tt2A2 exhibits an amorphous nature according to the XRD analysis (Figure 4). According to Table 3, the peaks at 2Theta = 9.65, 10.28, and 46.06° indicate the components of T in agreement with Zhu et al. (Zhu et al., 2024). Thymol is represented by a very strong peak at 8.38°, medium sized peaks at 7.7° and 11.40°, and weak peaks at 11.97° and 21.69° in comparison to previous investigations (Table 4) (Edis et al., 2024a; Edis et al., 2024b; Sharma et al., 2023). Few weak peaks indicate the presence of A in accordance to Bajpai et al. at 2Theta values of 13.45, 21.0, 33.76° and 62.25° (Table 4) (Bajpai et al., 2017).

### 3.2 Determination of antimicrobial activity

A detailed analysis of antimicrobial properties required testing varying thymol concentrations within the title formulation Tt2A2. The most effective two formulations were represented in the following discussions. The thymol-rich formulation was called Tt2A2^b^ and showed interesting results compared to the basic composition Tt2A2^a^. Both formulations inhibited an array of Gram-negative and Gram-positive bacteria.

Tea tree oil and thymol remain highly volatile, poorly soluble in water, therefore reduced bioavailability, susceptible to oxidative degradation and have dose-dependent toxicity (Burt, 2004; Carson et al., 2006; Kairey et al., 2023; Scientific Committee on Consumer Safety, 2025). However, oxidative degradation and sensitivity issues can be resolved by proper storage conditions at low temperatures in darkness and airtight containers, while production methods must be standardized (European Medicines Agency, 2015; Scientific Committee on Consumer Safety, 2025).

Kairey et al. concluded in their systematic review of randomized clinical trials inadequate evidence for translation into clinical practice and adverse effects at concentrations higher than 25% (Kairey et al., 2023). Additionally, Kairey et al. stress the need for novel formulations to improve local delivery of active ingredients (Kairey et al., 2023).

Accordingly, the title formulations in this study Tt2A2 are designed far below those critical concentration thresholds and are mixed with amoxicillin to support the antimicrobial action. The storage conditions did allow a shelf life of at least 3 months, but degraded after 18 months of same storage conditions.

Tea tree oil and thymol are recognized as medicinal substances within different regulatory frameworks which also address standardization problems (European Medicines Agency, 2015; European Medicines Agency, 2023; Organization for Standardization, 2025). These standards are directed towards quality assurance, reproducibility and clinical safety. Tea tree oil quality depends on natural essential oil composition through plant genotype, but also, environmental and climatic factors, as well as cultivation methods, harvesting type and timing, as well as extraction processes (Carson et al., 2006; European Medicines Agency, 2015; Kairey et al., 2023). Following those standards through comprehensive quality control measures ensures reproducibility, as well as consistency in antimicrobial properties and safety (European Medicines Agency, 2015; Scientific Committee on Consumer Safety, 2025; European Medicines Agency, 2023 **)**. However, these barriers remain to be considered in essential oil based antimicrobial agents. Accordingly, the title formulation in this study was prepared using very low concentration of tea tree oil in combination with low-dosed thymol and amoxicillin to ensure reproducibility and safety issues.

Table 5 displays the results of the tests conducted to determine antimicrobial activity in zone of inhibition (ZOI) diameters.

**TABLE 5:** Antimicrobial activity determination through disc diffusion assay of biological replicates by measurement of ZOI (mm) of antibiotics (A*), Tt2A2^a^ (serial dilution experiments 1^a^, 4^a^), Tt2A2^a18^ (1^a18^ is sample 1^a^ after 18 months) and Tt2A2^b^ (1^b^).

Strain	A*	A*	95% CI for mean (mm)	1^a^	95% CI for mean (mm)	1^b^	95% CI for mean (mm)
*S. pyogenes* ATCC 19615	G	24.67 ± 0.58	23.23-26.10	**29.67** ± 0.58	28.23-31.10	21.0 ± 4.0	11.06-30.94
*E. faecalis* ATCC 29212	G	24.67 ± 0.58	23.23-26.10	**27.67** ± 4.04	17.63-37.71	20.50 ± 4.5	9.32-31.68
*S. pneumoniae* ATCC 49619	G	18.33 ± 0.58	16.90-19.77	**30.33 ±** 8.08	10.25-50.41	16.0 ± 1.0	13.52-18.48
*S. aureus* ATCC 25923	G	28.33 ± 0.58	26.90-29.77	23,67 ± 2.31	17.93-29.40	25.50 ± 0.50	24.26-26.74
*B. subtilis* WDCM 00003	G	21.33 ± 0.58	19.90-22.77	**21.0 ±** 5.20	8.09-33.91	13.67 ± 1.53	9.87-17.46
*C. albicans* WDCM 00054	NY	15.67 ± 0.57	14.23-17.10	19.33 ± 2.08	14.16-24.50	35.0 ± 0.0	35.0–35.0
*K. pneumoniae* WDCM 00097	G	29.33 ± 0.58	27.90-30.77	15.0 ± 0.0	15.0-15.0	15.67 ± 0.58	14.23-17.10
*E. coli* WDCM 00013	G	22.67 ± 0.58	21.23-24.10	14.67 ± 0.58	13.23-16.01	15.0 ± 0.0	15.0-15.0
*P. mirabilis* ATCC 29906	G	29.33 ± 0.58	27.90-30.77	29.0 ± 1.73	0	24 ± 1.0	21.52-26.48
*P. aeruginosa* WDCM 00026	G	22.33 ± 0.58	20.90-23.77	12.0 ± 1.73	7.70-16.30	0	0

* ^a,b^Discs of 6 mm were impregnated with samples Tt2A2^a^ and Tt2A2^b^ of concentrations 1^a^: 2 mL of 40 μg/mL, 4^a^: 2 mL of 5 μg/mL, and 1^b^: 2 mL of 80 μg/mL. Antibiotics (A*) as positive control NY: Nystatin (100 IU) and G: Gentamicin (30 µg/disc). 0 indicates resistance. Bold numbers are higher ZOI, than the comparative ZOI, of control A* and underlined ZOI, are indicative of good inhibition. The grey shaded area represents Gram-negative reference strains. Calculated by one-way-ANOVA, with *p* > 0.05.

Data are presented as mean ± SD from three independent replicate measurements. Data extracted after normality test.

Disc diffusion revealed that samples Tt2A2^a^ and Tt2A2^b^ are effective against all ten reference pathogens (Table 5). Especially *P. mirabilis* ATCC 29906 and *P. aeruginosa* WDCM 00026 are usually resistant to multiple drugs due to their motility, an ability provided by their flagellae and further virulence factors (Edis et al., 2024a; Gan et al., 2023; Sánchez García et al., 2024). Remarkably, the formulation Tt2A2^a^ inhibited the growth of the motile strains of *P. mirabilis* ATCC 29906, *E. coli* WDCM 00013 and *P. aeruginosa* WDCM 00026 with ZOI of 29.0 ± 1.73 mm, 14.67 ± 0.58 mm and 12.0 ± 1.73 mm, respectively (Table 5). Furthermore, formulation Tt2A2^b^ showed inhibition zones for the same pathogens of 24.0 ± 1.0 mm, 15 ± 0 mm and 0 mm, respectively (Table 5). The unchanged antimicrobial and analytical results after storing the title formulations 3 months in darkness and fridge confirm their stability. Serial dilution studies revealed resistance and minimum inhibitory concentration for all Gram-negative pathogens at thymol concentrations of 5 μg/mL towards all Gram-negative reference strains, *C. albicans* WDCM 00054 and the Gram-positive *B. subtilis* WDCM 00003. Stability studies revealed changes in the formulations after 18 months of storage with resistance in 4 pathogens and reduced inhibitory action in the six other. The Gram-positive reference strains *S. pyogenes* ATCC 19615 and *E. faecalis* ATCC 29212, as well as the Gram-negative reference strains *P. mirabilis* ATCC 29906 and *P. aeruginosa* WDCM 00026 were no more susceptible to our formulation.

**FIGURE 5:**
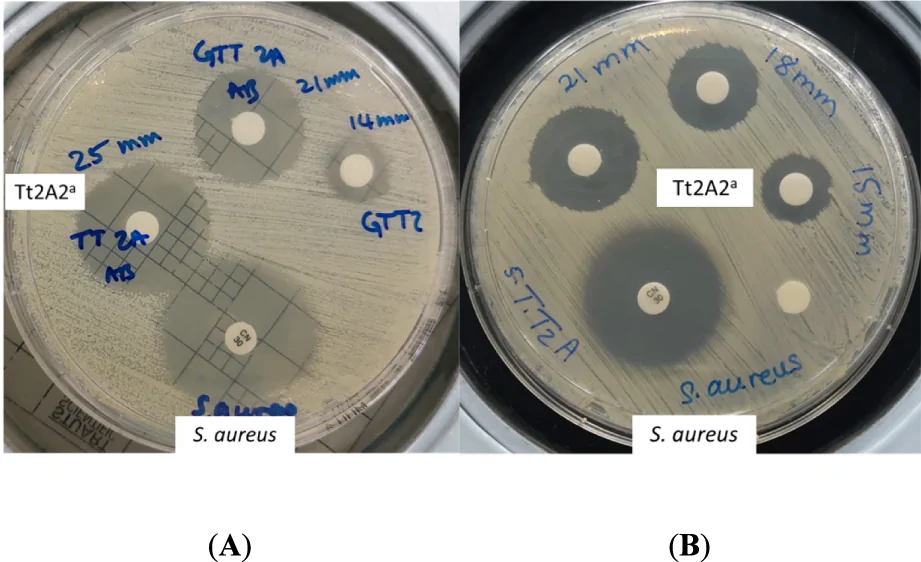
Tt2A2-coated sterile discs with positive control antibiotic gentamicin (30 µg/disc). From left to right **(A,B)**: *S. aureus* ATCC 25923.

Interestingly, Gram-positive reference strain *S. aureus* ATCC 25923 (Figure 5) and the fungi *C. albicans* WDCM 00054, which are both involved in dental caries and skin infections are inhibited by Tt2A2^a^ and Tt2A2^b^ at different concentrations (Table 5).

The results show a consistent pattern of pathogen inhibition on sterile discs for both formulations Tt2A2^a^ and Tt2A2^b^. Among the Gram-positive strains, *S. pneumoniae* ATCC 49619 was strongly inhibited by Tt2A2^a^ with a ZOI of 30.33 ± 8.08 mm compared to the positive control gentamycin with 18.33 ± 0.58 mm (Table 5). Furthermore, *S. pyogenes* ATCC 19615 and *E. faecalis* ATCC 29212 displayed strong ZOI of 29.67 ± 0.58 and 27.67 ± 4.04 mm in comparison to 24.67 ± 0.58 mm by gentamycin alone with Tt2A2^a^. The other formulation achieved similar results for both reference strains as well.

**FIGURE 6:**
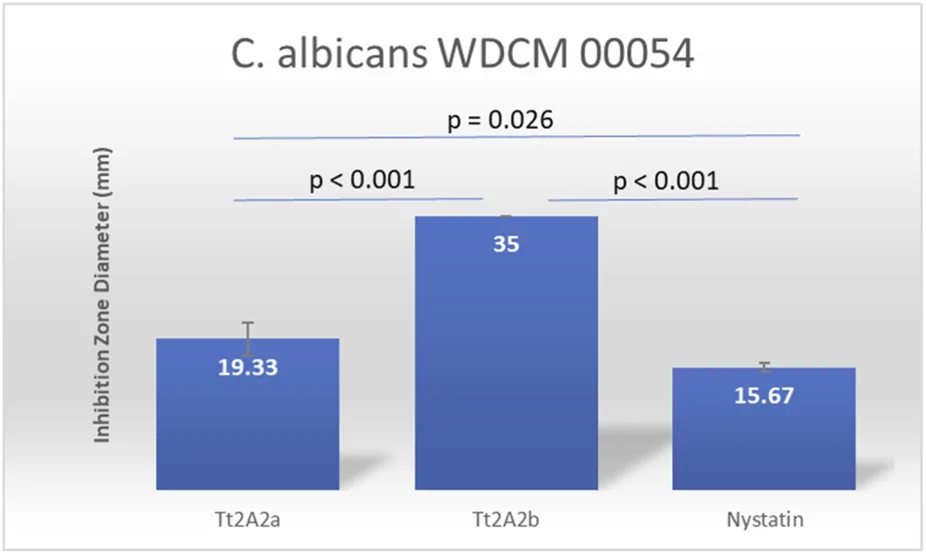
Antimicrobial activity of the tested formulations against C. albicans. Values are expressed as mean ± SD from triplicate measurements (n = 3). Error bars represent standard deviations. Statistical significance was assessed using one-way ANOVA followed by Tukey’s HSD *post hoc* test.

The pathogens known for transmission through surfaces by contact are *E. faecalis* ATCC 29212, *S. aureus* ATCC 25923, as well as the three motile bacilli *E. coli* WDCM 00013, *P. mirabilis* ATCC 29906, and *P. aeruginosa* WDCM 00026. There is almost no difference in ZOI between the two formulations Tt2A2^a^ and Tt2A2^b^ against the two Gram-positive bacteria cocci *E. faecalis* ATCC 29212 and *S. aureus* ATCC 25923. Nevertheless, the two Gram-negative *E. coli* WDCM 00013 and *P. mirabilis* ATCC 29906 are better inhibited by Tt2A2^b^ even at lower concentrations (Table 5). However, *P. aeruginosa* WDCM 00026 shows better inhibition when exposed to the formulation Tt2A2^a^ with ZOI = 12.0 ± 1.73 mm (Table 5). These results indicate different cell-membrane properties of the various strains and their degree of interaction leading to inhibition by Tt2A2^a^ and Tt2A2^b^. Table 6 displays one-way ANOVA Tests of Homogeneity of Variances, Tukey Post-Hoc Test with Multiple comparisons (significant difference for p < 0.05). The results reveal several significant differences in ZOI between selected formulations and the two antibiotic positive controls for each of the 10 reference strains. The p-values for the pairwise comparisons indicate following trends. The fungal strain *C. albicans* WDCM 00054 (Figure 6) and the Gram-Negative strain *P. aeruginosa* WDCM 00026 show in all comparisons between the formulations Tt2A2^a^, Tt2A2^b^ and the antibiotic positive controls significant differences in ZOI (one-way ANOVA and Tukey’s *post hoc* analysis both with p < 0.05). The Gram-negative reference strains except *P. mirabilis* ATCC 29906 reveal a significant difference between both the formulations T2A2a and T2A2b with gentamycin. No significant difference in ZOI is observed between Tt2A2^a^ and Tt2A2^b^ for *K. pneumoniae* WDCM 00097 (Tukey’s *post hoc* analysis p = 0.269) and *E. coli* WDCM 00013 (Tukey’s *post hoc* analysis p = 0.679). Most of the Gram-positive reference strains exhibited no significant differences in ZOI between the groups. However, Tt2A2^a^ differed significantly from Tt2A2^b^ in the case of *S. pyogenes* ATCC 19615 (Tukey’s *post hoc* analysis p = 0.01) and *S. pneumoniae* ATCC 49619 (Tukey’s *post hoc* analysis p = 0.023). There was a significant difference between Tt2A2^a^ and the antibiotic positive control gentamycin for *S. aureus* ATCC 25923 (Tukey’s *post hoc* analysis p = 0.015), while the other comparisons did not differ significantly.

**TABLE 6:** One-way ANOVA Tests of Homogeneity of Variances, Tukey Post-Hoc Test with Multiple comparisons (significant difference for p < 0.05).

References strain	Comparison	Mean difference	Standard error	p-value	Significance
*S. pyogenes* ATCC 19615	Tt2A2^a^ vs. Tt2A2^b^	8.67	1.92	0.010	Significant
​	Tt2A2^a^ vs. G	5.0	1.92	0.090	Not Significant
​	Tt2A2^b^ vs. G	−3.67	1.92	0.217	Not Significant
*E. faecalis* ATCC 29212	Tt2A2^a^ vs. Tt2A2^b^	7.17	2.86	0.102	Not Significant
​	Tt2A2^a^ vs. G	3.0	2.86	0.577	Not Significant
​	Tt2A2^b^ vs. G	−4.17	2.86	0.375	Not Significant
*S. pneumoniae* ATCC 49619	Tt2A2^a^ vs. Tt2A2^b^	14.33	3.85	0.023	Significant
​	Tt2A2^a^ vs. G	12.0	3.85	0.047	Significant
​	Tt2A2^b^ vs. G	−2.33	3.85	0.822	Not Significant
*S. aureus* ATCC 25923	Tt2A2^a^ vs. Tt2A2^b^	−1.83	1.15	0.316	Not Significant
​	Tt2A2^a^ vs. G	−4.67	1.15	0.015	Significant
​	Tt2A2^b^ vs. G	−2.83	1.15	0.106	Not Significant
*B. subtilis* WDCM 00003	Tt2A2^a^ vs. Tt2A2^b^	7.33	2.57	0.065	Not Significant
​	Tt2A2^a^ vs. G	−0.33	2.57	0.991	Not Significant
​	Tt2A2^b^ vs. G	−7.67	2.57	0.055	Not Significant
*C. albicans* WDCM 00054	Tt2A2^a^ vs. Tt2A2^b^	−15.67	1.02	<0.001	Significant
​	Tt2A2^a^ vs. NY	3.67	1.02	0.026	Significant
​	Tt2A2^b^ vs. NY	19.33	1.02	<0.001	Significant
*K. pneumoniae* WDCM 00097	Tt2A2^a^ vs. Tt2A2^b^	−0.67	0.39	0.269	Not Significant
​	Tt2A2^a^ vs. G	−14.33	0.39	<0.001	Significant
​	Tt2A2^b^ vs. G	−13.33	0.39	<0.001	Significant
*E. coli* WDCM 00013	Tt2A2^a^ vs. Tt2A2^b^	−0.33	0.39	0.679	Not Significant
​	Tt2A2^a^ vs. G	−8.0	0.39	<0.001	Significant
​	Tt2A2^b^ vs. G	−7.67	0.39	<0.001	Significant
*P. mirabilis* ATCC 29906	Tt2A2^a^ vs. Tt2A2^b^	0.67	4.39	0.987	Not Significant
​	Tt2A2^a^ vs. G	−4.67	4.39	0.568	Not Significant
​	Tt2A2^b^ vs. G	−5.33	4.39	0.488	Not Significant
*P. aeruginosa* WDCM 00026	Tt2A2^a^ vs. Tt2A2^b^	12.0	0.86	<0.001	Significant
​	Tt2A2^a^ vs. G	−10.33	0.86	<0.001	Significant
​	Tt2A2^b^ vs. G	−22.33	0.86	<0.001	Significant

Gram-Negative strains are shaded with gray.

In our previous study on AV-PVP-Thyme-I_2_ and AV-PVP-Thymol-I_2_, lower thymol concentrations (33 μg/mL) allowed iodine release through synergistic action with Aloe Vera biocomponents within the polymer polyvinylpyrrolidone (PVP) (Edis et al., 2024a; Edis et al., 2024b). AV-PVP-Thyme-I_2_ and AV-PVP-Thymol-I_2_ exhibited strong antifungal activity against *C. albicans* WDCM 00054 with 61 mm ZOI in comparison to 21 mm 15 mm by Tt2A2^b^ and Tt2A2^a^, respectively (Edis et al., 2024a). In conclusion, the susceptibility of *C. albicans* WDCM 00054 is in reverse order to the thymol concentration. The lowest thymol concentration of 33 μg/mL achieved the highest inhibition zone of 61 mm in the two PVP complexed iodinated-AV formulations (Edis et al., 2024a; Edis et al., 2024b). Meanwhile, the highest thymol concentration of 80 μg/mL showed ZOI of 15 mm in Tt2A2^b^. Interestingly, a previous investigation by Zhou et al. with pure thymol (100 μg/mL) against *S. aureus* strains reported the development of resistance after 30 passages with thymol (Zhou et al., 2019). The process was explained by cell membrane permeability of thymol resulting in depletion of NADPH at even concentrations of 400 μg/mL (Zhou et al., 2019). However, exploring combinations to lower the thymol concentration would prevent the development of bacterial resistance (Edis et al., 2024a).

In comparison, Tt2A2^a^, Tt2A2^b^ and AV-PVP-Thymol-I_2_ exhibit higher antimicrobial activity towards Gram-positive reference strains than Gram-negative (Table 5) (Edis et al., 2024a; Edis et al., 2024b; Zhou et al., 2019).

Table 7 compares the positive control antibiotics with amoxicillin against the 10 reference strains. For the analysis of amoxicillin with n = 4 different biological samples, Tukey’s HSD analysis divided the reference strains into three homogeneous subsets (Table 7). *Candida albicans* WDCM 00054, *K. pneumoniae* WDCM 00097 and *P. aeruginosa* WDCM 00026 displayed no antimicrobial activity (ZOI = 0 mm) and belonged to the lowest subset. *Bacillus subtilis* WDCM 00003, *E. coli* WDCM 00013, and *S. pneumoniae* ATCC 49619 formed an intermediate subset (14.5–20.5 mm), whereas *P. mirabilis* ATCC 29906, *S. pyogenes* ATCC 19615, *E. faecalis* ATCC 29212, and *S. aureus* ATCC 25923 exhibited the largest inhibition zones (23.0–28.0 mm) and belonged to the highest subset.

**TABLE 7:** Antimicrobial activity determination through disc diffusion assay by measurement of ZOI (mm) of antibiotics (A*), amoxicillin (A), thymol (t), T, mixture of T and t (1:2), Tt2A2 (1^a^ and 1^b^), and terpinene-4-ol (T-4-ol). Data are presented as mean ± SD from three independent replicate measurements. Data extracted after normality test. Results of selected testing results for comparison in ZOI [mm].

Strain	A*	A*	95% CI for mean (mm)	A	95% CI for mean (mm)
*S. pyogenes* ATCC 19615	G	24.67 ± 0.58	23.23-26.10	24.0 ± 1.15	22.16-25.84
*E. faecalis* ATCC 29212	G	24.67 ± 0.58	23.23-26.10	26.25 ± 3.20	21.16-31.34
*S. pneumoniae* ATCC 49619	G	18.33 ± 0.58	16.90-19.77	20.5 ± 4.04	14.07-26.93
*S. aureus* ATCC 25923	G	28.33 ± 0.58	26.90-29.77	28.0 ± 6.0	18.45-37.55
*B. subtilis* WDCM 00003	G	21.33 ± 0.58	19.90-22.77	14.50 ± 0.58	13.58-15.42
*C. albicans* WDCM 00054	NY	15.67 ± 0.57	14.23-17.10	0	0
*K. pneumoniae* WDCM 00097	G	29.33 ± 0.58	27.90-30.77	0	0
*E. coli* WDCM 00013	G	22.67 ± 0.58	21.23-24.10	20.25 ± 0.50	19.45-21.05
*P. mirabilis* ATCC 29906	G	29.33 ± 0.58	27.90-30.77	23.0 ± 6.27	13.02-32.98
*P. aeruginosa* WDCM 00026	G	22.33 ± 0.58	20.90-23.77	0	0

* Discs of 6 mm were impregnated with 2 mL of 1.37 μg/mL amoxicillin (A). Antibiotics (A*) as positive control NY: Nystatin (100 IU) and G: Gentamicin (30 µg/disc). 0 indicates resistance. No statistically significant differences with *p* > 0.05 by one-way ANOVA.

The high inhibitory action of Tt2A2^a^ against the selection of 10 pathogens made the formulation eligible for screening on masks, bandages (cotton gauze) and surgical sutures (Table 8).

**TABLE 8:** Antimicrobial activity determination of Tt2A2^a^ through disc diffusion assay by measurement of ZOI (mm) of antibiotics (A*), KN95 mask (KN95^a^), surgical face mask white layer (M_W_^a^), surgical face mask blue layer (M_B_^a^), cotton bandage (B^a^) and surgical suture (S^a^). Results of exemplary biofunctionalized Tt2A2^a^ on the same day of testing with the same fresh cultures for comparison in ZOI [mm].

Strain	A*	KN95^a^	M_W_^a^	M_B_^a^	B^a^	S^a^	t	T	Tt2	T-4-ol
*S. pyogenes* ATCC 19615	25	14	11	8	12	0	29	10	20	0
*E. faecalis* ATCC 29212	25	15	12	9	9	0	23	10	15	0
*S. pneumoniae* ATCC 49619	18	14	10	8	10	0	**0**	8	17	0
*S. aureus* ATCC 25923	28	14	10	7	11	0	43	11	15	0
*B. subtilis* WDCM 00003	21	10	8	3	8	0	40	10	18	0
*C. albicans* WDCM 00054	16	22	8	4	16	0	45	10	23	0
*K. pneumoniae* WDCM 00097	30	14	6	3	6	0	39	11	17	7
*E. coli* WDCM 00013	23	12	10	8	5	0	34	15	13	7
*P. mirabilis* ATCC 29906	30	14	15	7	14	0	**0**	0	0	0
*P. aeruginosa* WDCM 00026	23	3	3	1	2	0	**0**	0	12	0

* ^a^ Biomedical tools impregnated with 2 mL of 20 μg/mL of Tt2A2^a^: KN95^a^ face mask, M_W_^a^: white layer of surgical mask, M_B_^a^: blue layer of surgical mask, B^a^: bandage, S^a^: suture (all resistant). Discs of 6 mm were impregnated with 2 mL: 40 μg/mL of sample, thymol (t), T and thymol 1:2 mixture (Tt2and terpinene-4-ol (T-4-ol). Antibiotics (A*) as positive control NY: Nystatin (100 IU) and G: Gentamicin (30 µg/disc). 0 indicates resistance. The grey shaded area represents Gram-negative reference strains. The numbers represent screening with n = 1.

Table 8 indicates interesting patterns of microbial inhibition depending on biomedical material, type of pathogen and thymol concentration. In general, passive biomedical tissues can be changed to a bioactive material by impregnating them with the formulations on wound bandages, sutures and masks (Edis et al., 2024a; Edis et al., 2024b; Ferraz, 2025; Garapati et al., 2023; Makrygiannis et al., 2025; Szabelski and Karpiński, 2024). Accordingly, infection control measures could be enhanced by employing the title formulations as barrier on the above mentioned materials (Edis et al., 2024a; Edis et al., 2024b; Ferraz, 2025; Garapati et al., 2023; Makrygiannis et al., 2025; Szabelski and Karpiński, 2024).

Impregnating KN95 or surgical face mask tissues could prevent transmission into or out of the respiratory system. Furthermore, inhaling the formulations on the mask during infection could mitigate inflammatory processes leading to secondary microbial infection, as well as pathogen proliferation in the oral and nasal cavities (Edis et al., 2024a; Edis et al., 2024b).

The two Gram-positive strains *S. pneumoniae* and *S. aureus* are known to be transferred by air droplets into the respiratory system and could be inhibited before entering the oral- and nasal cavities (Edis et al., 2024a; Edis et al., 2024b). Remarkably, on KN95 face mask tissues, formulation Tt2A2^a^ reveals inhibition zones of 14 mm against *S. pneumoniae* ATCC 49619 and *S. aureus* ATCC 25923 (Table 6).

**FIGURE 7:**
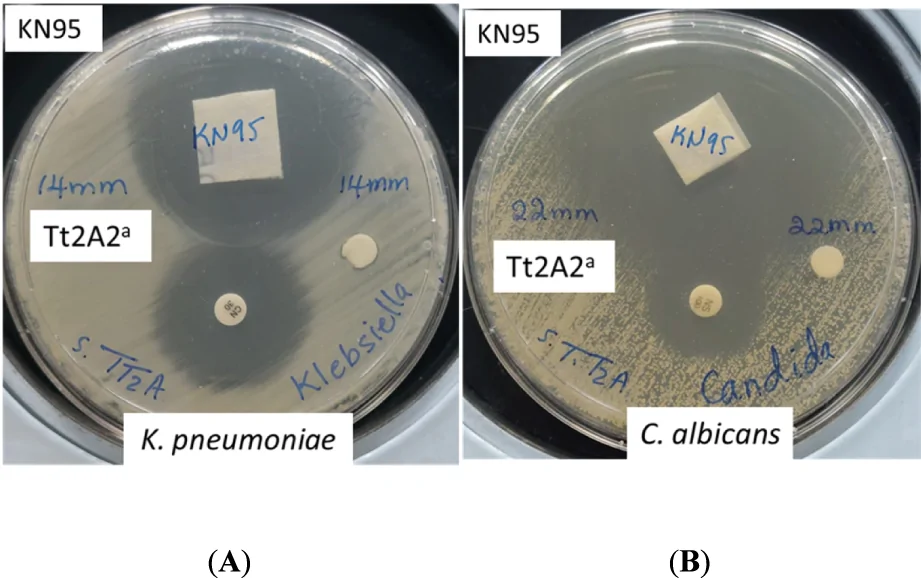
Tt2A2-coated on sterile KN95 mask tissue with positive control antibiotics nystatin (100 IU) and gentamicin (30 µg/disc). From left to right: Tt2A2^a^ (40 μg/mL): **(A)** *Klebsiella pneumoniae* WDCM 00097 and **(B)** *Candida albicans* WDCM 00054.

Figure 7 shows examples of KN95 mask tissues coated with the title formulations against different pathogens. *Candida albicans* WDCM 00054 and *K. pneumoniae* WDCM 00097 are inhibited by the formulation Tt2A2^a^ with 22 and 14 mm, respectively (Table 6; Figure 6). These two pathogens have partly hydrophilic outer cell membranes and further characteristics, which do not result in higher inhibition zones to higher hydrophobic thymol concentration in Tt2A2^b^ (Edis et al., 2024a; Edis et al., 2024b).

**FIGURE 8:**
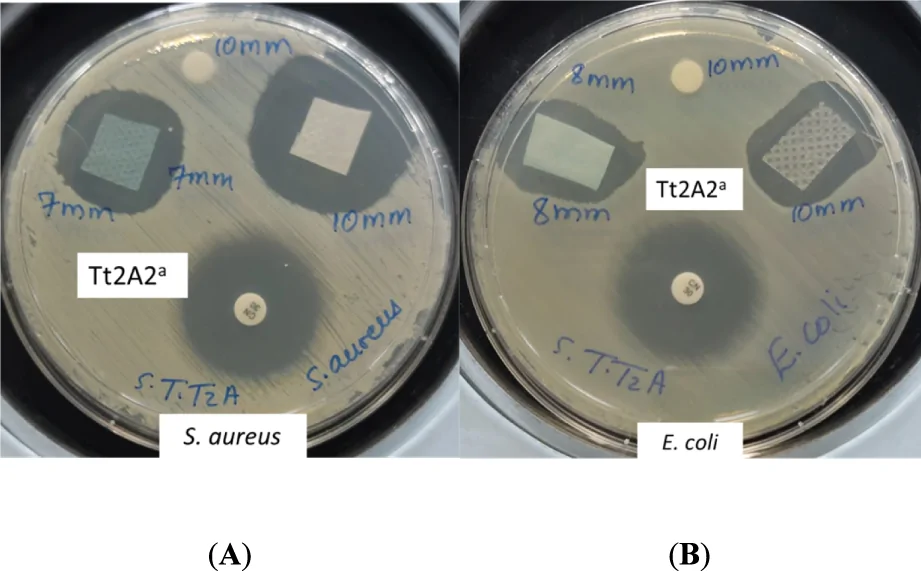
Tt2A2-coated on sterile 2-ply non-woven surgical mask tissues with positive control antibiotics gentamicin (30 µg/disc). From left to right: Tt2A2^a^ (40 μg/mL): **(A)** *S. aureus* ATCC 25923 and **(B)** *E. coli* WDCM 00013.

Figure 8 displays 2-ply, polypropylene disposable, non-woven surgical facemask fabrics layers (blue and white) coated with the title formulations against *S. aureus* ATCC 25923 and *E. coli* WDCM 00013. The formulations are clearly more antimicrobial on the white surgical mask layer, than the blue layer. The outer blue layer of a 2-ply non-woven facemask is hydrophobic and harder, while the white, inner fabric is softer and water-absorbent. The hydrophobic components T and thymol are therefore more adsorbed into the blue, water-repellent, chemically treated layer. This happens especially in the formulation Tt2A2^a^ with the lower thymol concentration.

**FIGURE 9:**
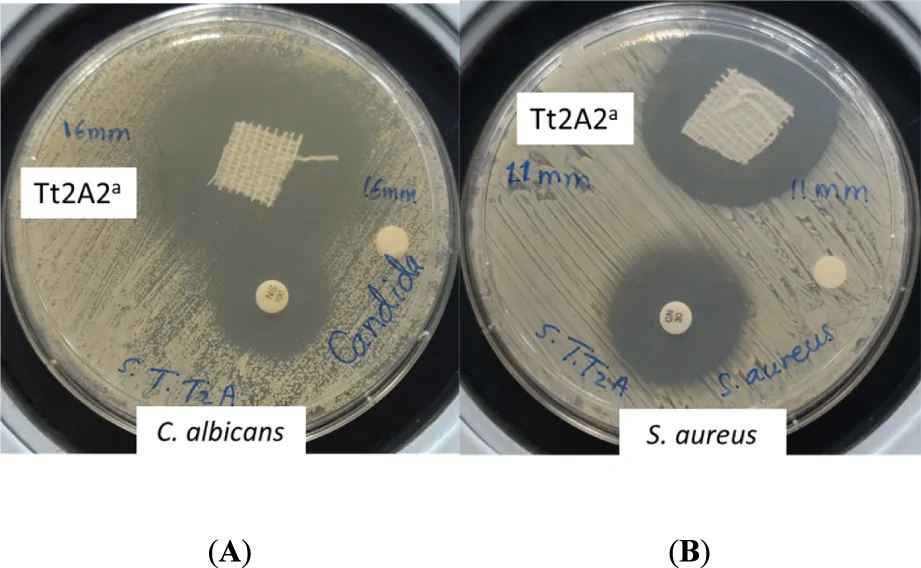
Tt2A2-coated on sterile cotton bandages with positive control antibiotics nystatin (100 IU) and gentamicin (30 µg/disc). From left to right: sterile cotton bandages with Tt2A2^a^ (40 μg/mL): **(A)** *Candida albicans* WDCM 00054 and **(B)** *S. aureus* ATCC 25923.

Cotton gauze bandages and surgical sutures are important in wound closure and healing. Wound- and surgical site infections (SSI) are a major concern in bacterial biofilm formation, delay or disruption of healing processes and introducing especially nosocomial infections by multidrug-resistant strains like *S. aureus*. Coating cotton gauzes and surgical sutures with antimicrobial compounds aids wound closure. This biofunctionalization not only induces rapid wound closure but also prevention of microbial proliferation on the tissues and the biomaterial itself (Edis et al., 2024a; Edis et al., 2024b; Ferraz, 2025; Garapati et al., 2023; Makrygiannis et al., 2025; Szabelski and Karpiński, 2024). Impregnating antimicrobial formulations into the biomaterials renders them from passive to bioactive by reducing or preventing bacterial contamination, reducing the need for wound care and promoting healing according to Ferraz et al. (Ferraz, 2025). We coated sterile cotton gauze and surgical sutures with the title formulation Tt2A2^a^ (Table 8; Figure 9).

The formulation Tt2A2^a^ inhibits *C. albicans* WDCM 00054 and *S. aureus* ATCC 25923 with 16 and 11 mm on sterile cotton bandages, respectively (Table 8; Figure 9). On sterile cotton bandages, *C. albicans* WDCM 00054 is inhibited by the formulation Tt2A2^a^ more than all the other reference strains (Table 8). Recently, Buriti et al. reported about the effects of the wound-healing properties of thymol, carvacrol, and eugenol against bacteria on skin and confirmed above results (Buriti et al., 2024).

The utilized absorbable PGA sutures are hydrophilic and strongly adsorbed the formulation Tt2A2^a^ (Table 8). Therefore, all reference strains were not inhibited.

In conclusion, *C. albicans* WDCM 00054 is susceptible to the formulation with less thymol Tt2A2^a^ on the biofunctionalized cotton bandages, KN95, surgical face mask and the sterile discs (Table 8). The reason is the negatively charged, hydrophilic outer cell wall with mannoproteins, which are easily penetrated and destroyed by Tt2A2^a^.

The results are in agreement with our previous formulations AV-PVP-Thyme-I_2_ and AV-PVP-Thymol-I_2_, although the ZOI in bandages and masks are slightly lower in this study (Edis et al., 2024a; Edis et al., 2024b).

Further detailed investigations on the formulations antimicrobial properties were conducted to decide, which one of the components or combinations actually inhibit the reference strains. Table 8 compares thymol (t), T, a 1:2 mixture between T and T (Tt2), terpinene-4-ol (T-4-ol) by disc-diffusion methods. As a basic study, our results show potential in mixing ingredients, and may be used to justify formal synergy studies in future work.

The formulations appear to outperform individual components. Similarly, the performance of the formulations Tt2A2 is comparable to that of the positive controls gentamycin and nystatin. Although resisted by certain strains, standalone thymol was able to inhibit the rest to a stronger extent in comparison to the positive controls, with the exception of *E. faecalis*. When combined with the other ingredients, all strains were inhibited albeit to lesser extents.

The first details arise by noticing which pathogens are resistant towards the compounds. Amoxicillin does not stop microbial proliferation of *C. albicans* WDCM 00054, *K. pneumoniae* WDCM 00097 and *P. aeruginosa* WDCM 00026 (Table 7).

*Pseudomonas aeruginosa* WDCM 00026, *P. mirabilis* ATCC 29906 and *S. pneumoniae* ATCC 49619 are resistant towards thymol (Table 8).

T is inactive against *P. aeruginosa* WDCM 00026 and *P. mirabilis* ATCC 29906. The combination Tt2 is only unable to inhibit *P. mirabilis* ATCC 29906 (Table 8).

Pure terpinene-4-ol, which is the major ingredient in T is only able to weakly inhibit *K. pneumoniae* WDCM 00097 and *E. coli* WDCM 00013 with ZOI = 7 mm. All the other reference strains are resistant against T-4-ol. This is clearly in opposition to previously reported results of antimicrobial activities of terpinene-4-ol in the literature (Cordeiro et al., 2020; Ferreira et al., 2023; Francisconi et al., 2020; Johnson et al., 2022; Kamel et al., 2022; Yuan et al., 2025). The last result underlines the importance of synergistic biocomponents within natural T. These components ameliorate the antimicrobial properties of T in comparison to T-4-ol (Table 8).

However, T alone only displays weak inhibitory action on most of the selected strains, while two of them remain resistant. These are the two motile Gram-negative pathogens *P. aeruginosa* WDCM 00026 and *P. mirabilis* ATCC 29906 (Table 8). Recently, Sánchez García et al. confirmed this finding (Sánchez García et al., 2024). They reported motility inhibition of *P. vulgaris, E. coli, and P. aeruginosa* by a thymol-T-terpinene-4-ol formulation, while the pure compounds failed to achieve the same effect on their own (Sánchez García et al., 2024).

The mixture between one part T and two parts thymol (Tt2) achieves better antimicrobial results through additive effects against *S. pneumoniae* ATCC 49619 and *P. aeruginosa* WDCM 00026 (Table 8). Tt2, T and t are all unable to inhibit *P. mirabilis* ATCC 29906 (Table 8). Antagonistic effects were observed towards *S. aureus* ATCC 25923, *B. subtilis* WDCM 00003, *C. albicans* WDCM 00054, *K. pneumoniae* WDCM 00097 and *E. coli* WDCM 00013 (Table 8). In these cases, the very high inhibitory action of thymol was compromised by the addition of T to intermediate and weak results (Table 8).

Adding two parts of amoxicillin into Tt2 improves the antiproliferative action of Tt2 in most of the reference strains except those which are originally resistant towards amoxicillin itself (Table 8). All the reference strains are inhibited by the formulations Tt2A2^a^ and Tt2A2^b^ at different levels.

In general, the formulations Tt2A2^a^ and Tt2A2^b^ are inhibiting cocci/Gram-positive pathogens better than *C. albicans* WDCM 00054 and bacilli/Gram-negative pathogens in descending order (Table 5). An interesting exception is the high antimicrobial action against the swarming *P. mirabilis* ATCC 29906 with numerous flagellae.

Furthermore, amongst Gram-positive pathogens there is a pattern related to aggregation. Diplococci (*S. pneumoniae* ATCC 49619) are inhibited highly, followed by chains (*S. pyogenes* ATCC 19615, *E. faecalis* ATCC 29212), followed by complex aggregate *S. aureus* ATCC 25923 in descending order (Tables 5, 8).

As the thymol (t) concentration increased in Tt2A2^b^, diameter of ZOI increased as well. This proportionality is vital when employing sample Tt2A2 in practical application. Thymol, as a constituent of thyme and other plants, has shown immense potential for antimicrobial activity (Edis et al., 2024a; Edis et al., 2024b; Gago et al., 2025; Giotopoulou et al., 2024; Kowalczyk et al., 2020; Marchese et al., 2016; Meeran et al., 2017; Parolin et al., 2024; Sánchez García et al., 2024; Sharma et al., 2023; Zamani et al., 2015; Zhou et al., 2019; Đorđević et al., 2025). Moreover, thymol and its isomer carvacrol are known to destroy the outer membrane of bacteria and increase its fluidity (Dong et al., 2024; Hajibonabi et al., 2023).

In the context of the biomedical tools, impregnation of surgical sutures showed no effect to any of the tested pathogens for Tt2A2^a^ (Table 8).

**FIGURE 10:**
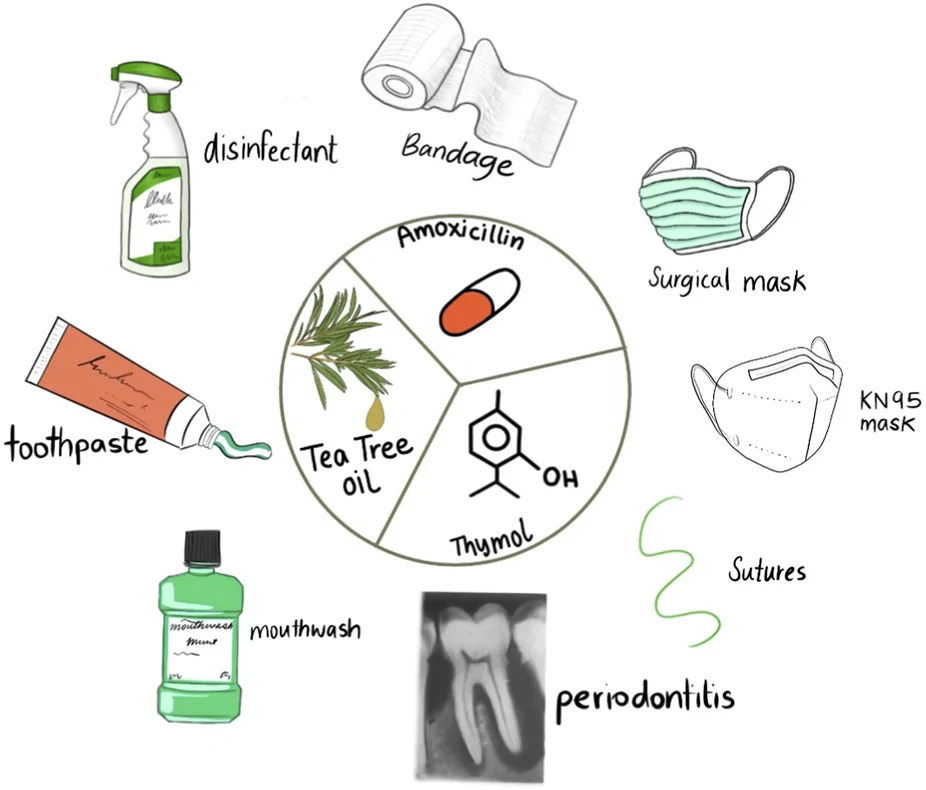
Tt2A2 and potential applications.

The formulations Tt2A2^a^ and Tt2A2^b^ can be utilized for oral care and dental treatment. Oral pathogens like the Gram-positive *S. aureus*, *E. faecalis*, *S. pyogenes*, and the Gram-negative *E. coli*, which colonize and form biofilms in oral cavities around teeth, gingiva and dentin walls of root canals (Brożyna et al., 2021). Especially *E. faecalis* is responsible for progression from colonization, to biofilm formation, then inflammation, plaque formation to dental caries, finally periodontal disease and further possible systemic diseases. Furthermore, *S. pyogenes* is the leading cause from starting dental plaque towards pneumonia, endocarditis, or encephalitis. These opportunistic microorganisms could therefore be prevented in the formation of SSI (Edis et al., 2024b; Maggio et al., 2025). The use of the title formulations within mouthwash or tooth paste could ameliorate pathogen proliferation and biofilm formation in the oral cavity (Edis et al., 2024b). Biofunctionalization of Tt2A2^a^ could therefore be possible on cotton bandages, face masks, both of the KN95 type and the surgical type, on discs, as disinfectant spray for surface areas, and mouthwash or even tooth paste ingredient (Figure 10).

The antimicrobial results on bandages, sutures and face masks highlight the potential of the formulation, which could be used in wound care and control of air-droplet transmission of microorganisms (Table 8). Simultaneously, before direct application, biocompatibility and safety must be considered (Rosa et al., 2024). Further work is required in the form of MBC, MIC, MBF studies, as well as cytotoxicity assays, hemolysis assays, and skin irritation studies.

Formulations Tt2A2^a^ and Tt2A2^b^ warrant further study, highlighting the significance of the investigated applications with their promising outcomes (Ferreira et al., 2023; Johnson et al., 2022; Kamel et al., 2022). The formulations were prepared by simple one-pot-synthesis without the techniques of nanoemulsion, nanoencapsulation or polymerization to eliminate the need for any further additive or detrimental preparation method. Instead, thymol-concentration in both formulations governs the antimicrobial activities. In general, low-dose amoxicillin (A) combined with low dosed T and thymol ensured good antimicrobial results. This could be a step forward against AMR by reducing the use of antibiotics. The low concentration of thymol and T in the formulation may have reduced their disadvantages and increased their effectivity, stability and delivery. Future investigations on the concentration dependence and cytotoxicity are warranted to confirm the use of biofunctionalized applications. Likewise, for confirmation of antimicrobial potential, determination of other quantitative microbial assessments e.g., minimum inhibitory concentration, minimum bactericidal concentration, and minimum fungicidal concentration, as well as *in vivo* antimicrobial properties are required.

The statistical analysis performed through One-way ANOVA, followed by Tukey’s *post hoc* analysis shows that the observed differences are highly reproducible. The one-way ANOVA demonstrated significant differences among the experimental formulations (Formulation Tt2A2^a^: F (9, 20) = 109.202 with p < 0.001 and Formulation Tt2A2^b^: F (9, 20) = 61.496 with p < 0.001). Tukey’s *post hoc* analysis was used to identify specific formulations responsible for the given differences. Additionally, the positive-control antibiotics also performed experimental reproducibility, as reflected by the very low within-group variance (mean square = 0.333) and a highly significant ANOVA result (F (9, 20) = 188.667 with p < 0.001). These findings reveal that the assay was performed consistently and that the observed differences among treatments are unlikely to be attributable to experimental variability. Our primary objectives of this investigation were designed to screen the tested formulations using widely disc diffusion and serial dilution methods on agar plates. Therefore, we compared antimicrobial activities of different thymol-tea-tree oil and amoxicillin formulations under identical experimental conditions. These were carried out by three biological replicates at different days with fresh samples through standardized methods, same reference strains, inoculum density and same incubation periods The standard agar plates purchased from Himedia were coded and measured in blinded manner minimizing observer bias. Employing standard agar plates ensured minimal difference in agar dept and concentration, as well as better performance throughout, including in the serial diffusion experiments (Table 5).

The statistical analysis by one-way ANOVA was performed to include normality and homogeneity of variance. Normality was calculated by the Shapiro-Wilk test, while the homogeneity was calculated by Levene’s test before performing ANOVA. The differences among the groups were analysed by one-way ANOVA, followed by Tukey’s *post hoc* test including a statistical significance of p < 0.05. The results verify the assumptions of the statistical analysis. The results indicate, that the assumptions were satisfied for the antibiotic dataset (Levene’s test, p = 1.000), while some unequal variances were seen for Tt2A2a (p < 0.001) and Tt2A2b (p = 0.0035) datasets. All ANOVA statistics are based on triplicate agar diffusion assays for each microbial strain and formulation with independent biological replicates in each group (n = 3). This methodology is considered reasonably robust with moderate violations of the homogeneity and normality assumptions.

For *P. aeruginosa* WDCM 00026 and *C. albicans* WDCM 00054, Tukey’s HSD analysis separated Tt2A2b, Tt2A2a, and the positive control antibiotic into three distinct homogeneous subsets, indicating significant pairwise differences among all treatments (p < 0.05) revealing significant differences among all variables. None of the variables (Tt2A2a, Tt2A2b, Gentamycin, Nystatin) share the same subset with each other. For *P. aeruginosa*, gentamycin positive control showed the largest ZOI of 22.33 mm, followed by Tt2A2a with 12 mm, while Tt2A2b showed did not have any inhibitory action ZOI = 0.00 mm. For *C. albicans* WDCM 00054, the largest ZOI with 35 mm is observed for Tt2A2b as the highest subset, followed by subset 2 and intermediate ZOI = 19.33 mm, then the lowest subset 1 with weak ZOI of 15.67 mm.

Tukey’s HSD *post hoc* analysis indicated that no significant pairwise differences were observed between Tt2A2a, Tt2A2b, and the gentamycin positive control (all p > 0.05) for *E. faecalis* ATCC 29212, *B. subtilis* WDCM 00003, *P. mirabilis* ATCC 29906.

*E. faecalis* ATCC 29212, Tt2A2a exhibited the largest mean inhibition zone (27.67 mm), followed by the gentamycin positive control (24.67 mm) and Tt2A2b (20.50 mm), Tukey's HSD test revealed no statistically significant differences among the treatments (p > 0.05). *B. subtilis* WDCM 00003 showed the largest ZOI with 21.33 mm for the gentamycin positive control, followed by Tt2A2a with 21.00 mm and lastly Tt2A2b with 13.67 mm. *P. mirabilis* ATCC 29906 exhibited the largest ZOI for gentamycin positive control (29.33 mm), followed by Tt2A2a (24.67 mm) and lastly Tt2A2b (24.0 mm).

Tukey’s HSD analysis grouped the treatments into two homogeneous subsets for *S. pyogenes* ATCC 19615, *S. pneumoniae* ATCC 49619, *S. aureus* ATCC 25923, *K. pneumoniae* WDCM 00097, *E. coli* WDCM 00013. The tested formulations (Tt2A2a and Tt2A2b) constituted one subset, while the gentamycin positive control formed a separate subset, reflecting significantly higher antimicrobial activity.

The Tukey’s HSD analysis for *S. pyogenes* ATCC 19615 and *S. aureus* ATCC 25923 resulted in two overlapping homogeneous subsets. In *S. pyogenes* ATCC 19615 with the gentamycin positive control, and in *S. aureus* ATCC 25923 with Tt2A2b appearing in both subsets, respectively, indicating that the treatment groups did not differ significantly at the 0.05 level.

For *K. pneumoniae*, Tukey’s HSD *post hoc* analysis showed no significant difference between Tt2A2a with ZOI = 15 mm and Tt2A2b ZOI = 15.67 mm, as both treatments belonged to the same homogeneous subset. However, the gentamycin positive control (29.33 mm) formed a separate higher subset and revealed significantly greater antimicrobial activity than both formulations Tt2A2.

The normality was violated (Tt2A2a and Gentamycin positive control) by all of the Gram-positive pathogens *S. pyogenes* ATCC 19615, *E. faecalis* ATCC 29212, *S. pneumoniae* ATCC 49619, *S. aureus* ATCC 25923, *B. subtilis* WDCM 00003 and the Gram-negative *P. mirabilis* ATCC 29906.

*C. albicans* WDCM 00054 violated the homogeneity in ANOVA between groups, in Tukey’s test (with Tt2A2a and Tt2A2b), while in post-hoc tests for multiple comparisons the normality was violated (Nystatin positive control). The Gram-negative *E. coli* WDCM 00013 and *P. aeruginosa* WDCM 00026 violated the homogeneity in ANOVA between groups, in Tukey’s test (with Tt2A2a and Tt2A2b), while in post-hoc tests for multiple comparisons the normality was violated (Tt2A2a and Gentamycin positive control). *K. pneumoniae* WDCM 00097 violated the homogeneity in ANOVA between groups, in Tukey’s test (with Tt2A2a and Tt2A2b), while in *post hoc* tests for multiple comparisons the normality was violated (Tt2A2b and Gentamycin positive control).

## 4 Conclusion

This investigation presents an exploratory *in-vitro* study screening antimicrobial properties of formulations between tea tree oil, thymol and amoxicillin against 10 selected reference strains in comparison to common antibiotics as positive controls. Therefore, there is no a priory power analysis, which is a methodological limitation. The investigation is based on standard practice in antimicrobial susceptibility methods based on agar diffusion assays and agar serial dilution methods by using standardized agar plates and procedures throughout. Therefore, this work is characterized as an early-stage screening study with the aim to identify feasible and effective biologically active formulations and their possible applications on biomedical tools. Future works based on the outcomes of this study need to test the presented hypothesis with predefined effect thresholds, MBC/MIC/MFC, toxicity, safety, *in-vivo*, pre-clinical and randomized clinical trials.

In this investigation, experimental validation was determined through three independent biological replicates and statistical analysis with one-way ANOVA, then by Tukey’s *post hoc* test, Shapiro-Wilk test for normality and finally Levene’s test for the homogeneity of variances. The Tukey’s *post hoc* test was used to determine the differences among the tested groups. The outcomes affirmed consistent difference between treatment groups, were discussed alongside observed ZOI and revealed relevant information about the inhibitory potency of the formulations. The antimicrobial testing was performed in triplicate with standard agar plates and procedures in alignment with common practice for disc diffusion assays. However, despite finding statistically significant differences, a formal power analysis was not conducted. Accordingly, future studies with larger sizes are guaranteed to increase reliable outcomes. All ANOVA statistics are based on triplicate agar diffusion assays for each microbial strain and formulation. These produced for each of the three groups df = 2 and df = 6 in the ANOVA. One-way ANOVA revealed significant differences among all three variables for C. albicans, F (2, 6) = 203.357, p < 0.001 and *P. aeruginosa* WDCM 00026 F (2,6) = 337.300, p < 0.001. There were no significant differences in all three comparisons for *E. faecalis* ATCC 29212 (F (2,6) = 3.158, p = 0.116), *B. subtilis* WDCM 00003 (F (2,6) = 5.697, p = 0.041) and *P. mirabilis* ATCC 29906 (F (2,6) = 0.877, p = 0.463). The other strains showed a mixture of significant and insignificant outcomes.

Several previous investigations are based on the combination of antibiotics with essential oils. Their antimicrobial results are varying and require further investigations. In this study, a novel, one-pot formulation of thymol, amoxicillin and T has been prepared, characterized, and tested against a selection of 10 reference microbial strains in alignment and comparison to previous investigations in our group. The investigative goal of this study was to compare the antimicrobial activities of Tt2A2 formulations under given conditions on discs and biomedical tools. The title formulation shows potential as antimicrobial agent, having demonstrated efficacy when used as a coating on face masks, gauze bandages, and especially PGA surgical sutures. Formulation Tt2A2^a^ revealed higher inhibition zones against most Gram-positive and some Gram-negative bacteria than Tt2A2^b^. However, formulation Tt2A2^b^ was more effective against *C. albicans* WDCM 00054. Limiting factors included deterioration of the formulation towards 18 months of storage and the standardization of the natural product T. The formulations showed resistance in our serial dilution studies at thymol concentrations of 5 μg/mL towards all Gram-negative reference strains, *C. albicans* WDCM 00054 and the Gram-positive *B. subtilis* WDCM 00003. Similarly, resistance appears after storage of 18 months in the Gram-positive reference strains *S. pyogenes* ATCC 19615 and *E. faecalis* ATCC 29212, as well as the Gram-negative reference strains *P. mirabilis* ATCC 29906 and *P. aeruginosa* WDCM 00026. Cytotoxicity studies and deeper quantitative antimicrobial assessments in future investigations are needed to firmly underscore the possible applications of Tt2A2. The statistical analysis revealed experimental reproducibility. In this study, the aim was to minimize the disadvantages of thymol and T by combining them at low concentrations with amoxicillin. Furthermore, the use of reduced amoxicillin concentrations in formulations supported by natural antimicrobials could be an effective tool against the ongoing AMR. The results reveal the option to fine-tune the composition of the title sample towards the targeted micro-organism and utilized biomaterial. Therefore, these one-pot-formulations with thymol, amoxicillin and T can be tailored and used selectively on biomedical tools against specific, distinctively chosen pathogens. The antimicrobial activities were directly dependent on the thymol concentration at a low concentration of amoxicillin, but not on T and terpinene-4-ol.

## Data Availability

The original contributions presented in the study are included in the article/Supplementary Material, further inquiries can be directed to the corresponding author.

## References

[B1] Abraha A. Kebede A. Belay A. (2016). Study the self-association of amoxicillin, thiamine and the hetero-association with biologically active compound chlorgenic acid. Afr. J. Pharm. Pharmacol. 10, 393–402. 10.5897/AJPP2016.4542

[B2] Ahmed S. K. Hussein S. Qurbani K. Ibrahim R. H. Fareeq A. Mahmood K. A. (2024). Antimicrobial resistance: impacts, challenges, and future prospects. J. Med. Surg. Public Health 2, 100081. 10.1016/j.glmedi.2024.100081

[B3] Akhtar M. F. Irshad M. Ali S. Summer M. Gulrukh S. Irfan M. (2024). Evaluation of biological potential of UV-spectrophotometric, SEM, FTIR, and EDS observed Punica granatum and Plectranthus rugosus extract-coated silver nanoparticles: a comparative study. Microsc. Research Technique 87 (3), 616–627. 10.1002/jemt.24454 38031715

[B4] Bajpai S. K. Shah F. F. Bajpai M. Jadaun M. Jyotishi P. (2017). Dynamic release of amoxicillin from orally dissolving film (ODF) composed of casein and sodium alginate. J. Drug Res. Dev. 3 (2). 10.16966/2470-1009.134

[B5] Bassolé I. H. N. Juliani H. R. (2012). Essential oils in combination and their antimicrobial properties. Molecules 17 (4), 3989–4006. 10.3390/molecules17043989 22469594 PMC6268925

[B6] Bauer A. W. Perry D. M. Kirby W. M. M. (1959). Single-disk antibiotic-sensitivity testing of staphylococci: an analysis of technique and results. AMA Arch. Intern. Med. 104, 208–216. 10.1001/archinte.1959.00270080034004 13669774

[B7] Bejar E. (2017). “Adulteration of tea tree oil (Melaleuca alternifolia and M. Linariifolia). Botanical adulterants prevention bulletin,” in ABC–AHP–NCNPR Botanical Adulterants Prevention Program (Austin, TX: American Botanical Council), 1–8.

[B8] Bertocchi M. Rigillo A. Elmi A. Ventrella D. Aniballi C. ; G. Scorpio D. (2020). Preliminary assessment of the mucosal toxicity of tea tree (Melaleuca alternifolia) and rosemary (rosmarinus officinalis) essential oils on novel porcine uterus models. Int. J. Mol. Sci. 21, 3350. 10.3390/ijms21093350 32397373 PMC7247571

[B9] Borotová P. Galovičová L. Vukovic N. L. Vukic M. Tvrdá E. Kačániová M. (2022). Chemical and biological characterization of Melaleuca alternifolia essential oil. Plants 11, 558. 10.3390/plants11040558 35214891 PMC8880210

[B10] Brożyna M. Paleczny J. Kozłowska W. Chodaczek G. Dudek-Wicher R. Felińczak A. (2021). The antimicrobial and antibiofilm *in vitro* activity of liquid and vapour phases of selected essential oils against *Staphylococcus aureus* . Pathogens 10, 1207. 10.3390/pathogens10091207 34578239 PMC8466273

[B11] Buriti B. M. A. d.B. Figueiredo P. L. B. Passos M. F. da Silva J. K. R. (2024). Polymer-based wound dressings loaded with essential oil for the treatment of wounds: a review. Pharmaceuticals 17, 897. 10.3390/ph17070897 39065747 PMC11279661

[B12] Burt S. (2004). Essential oils: their antibacterial properties and potential applications in foods—A review. Int. J. Food Microbiol. 94 (3), 223–253. 10.1016/j.ijfoodmicro.2004.03.022 15246235

[B13] Butucel E. Balta I. Bundurus I. A. Popescu C. A. Iancu T. Venig A. (2023). Natural antimicrobials promote the anti-oxidative inhibition of COX-2 mediated inflammatory response in primary oral cells In-fected with *Staphylococcus aureus*, Streptococcus pyogenes and *Enterococcus faecalis* . Antioxidants 12, 1017. 10.3390/antiox1205101 37237883 PMC10215671

[B14] Carson C. F. Hammer K. A. Riley T. V. (2006). *Melaleuca alternifolia* (tea tree) oil: a review of antimicrobial and other medicinal properties. Clin. Microbiol. Rev. 19 (1), 50–62. 10.1128/CMR.19.1.50-62.2006 16418522 PMC1360273

[B15] Cen C. Wang X. Li H. Miao S. Chen J. Wang Y. (2025). Nano-emulsification potentiates tea tree oil bioactivity: high-stability formulation for dual antimicrobial and antioxidant food preservation. Foods 14, 3405. 10.3390/foods14193405 41097575 PMC12523386

[B16] CLSI (2019). Performance Standards for Antimicrobial Susceptibility Testing. 29th ed., 39. Wayne, PA, USA: Clinical and Laboratory Standards Institute.

[B17] Cordeiro L. Figueiredo P. Souza H. Sousa A. Andrade-Júnior F. Medeiros D. (2020). Terpinen-4-Ol as an antibacterial and antibiofilm agent against *Staphylococcus aureus* . Int. J. Mol. Sci. 21, 4531. 10.3390/ijms21124531 32630600 PMC7350221

[B18] Cristea A.-G. Lisă E.-L. Iacob S. Dragostin I. Ștefan C. S. Fulga I. (2025). Antimicrobial smart dressings for combating antibiotic resistance in wound care. Pharmaceuticals 18, 825. 10.3390/ph18060825 40573221 PMC12196261

[B19] Das S. Prakash B. (2024). “Effect of environmental factors on essential oil biosynthesis, chemical stability, and yields,” in Plant Es-sential Oils: From Traditional to modern-day Application. Editors Prakash B. Dubey N. K. Freitas Brilhante de São José J. (Singapore: Springer Nature), 225–247. 978-981-9943-70-8.

[B20] Diebner H. H. Wallrafen A. M. Timmesfeld N. Rahmel T. Nowak H. (2025). The actual clinical situation ruthlessly exposes the challenge of rational care for nosocomial and community-acquired infections and requires Even more efforts for satis-factory antibiotic stewardship. Antibiotics 14, 561. 10.3390/antibiotics14060561 40558151 PMC12189429

[B21] Dong H. You Y. Wang N. Wang M. Song T. He Y. (2024). Development of amphipathic de-rivatives of thymol and carvacrol as potent broad-spectrum antibacterial agents. Eur. J. Med. Chem. 276, 116716. 10.1016/j.ejmech.2024.116716 39088997

[B22] Đorđević N. Cvetković K. Stanojević J. Karabegović I. Savić D. Cvetković D. (2025). Essential oil mixtures: enhanced antimicrobial and antioxidant activity. Antibiotics 14, 180. 10.3390/antibiotics14020180 40001423 PMC11851906

[B23] Edis Z. Bloukh S. H. (2024). Thymol, a monoterpenoid within polymeric iodophor formulations and their antimicrobial activities. Int. J. Mol. Sci. 25, 4949. 10.3390/ijms25094949 38732168 PMC11084924

[B24] Edis Z. Bloukh S. H. Sara H. A. Bloukh I. H. (2024). Green synthesized polymeric iodophors with thyme as antimicrobial agents. Int. J. Mol. Sci. 25, 1133. 10.3390/ijms25021133 38256211 PMC10815993

[B25] Enciso-Martínez Y. Barrios-Villa E. Ballesteros-Monrreal M. G. Navarro-Ocaña A. Valencia D. González-Aguilar G. A. (2025). Virulence and antibiotic resistance of aEPEC/STEC esche-Richia coli pathotypes with serotype links to Shigella boydii 16 isolated from irrigation water. Pathogens 14, 549. 10.3390/pathogens14060549 40559557 PMC12195767

[B26] European Medicines Agency (EMA) (2015). European Union Herbal Monograph on Melaleuca alternifolia (Maiden and Betche) Cheel, M. linariifolia Smith, M. dissitiflora F. Muell. And/Or Other Species of Melaleuca, Aetheroleum. EMA/HMPC/320930/2012. London: European Medicines Agency.

[B27] European Medicines Agency (EMA) (2023). Addendum to Assessment Report on Melaleuca alternifolia (Maiden and Betche) Cheel, Melaleuca linariifolia Smith, Melaleuca dissitiflora F. Muell. And/or Other Species of Melaleuca, Aetheroleum. EMA/HMPC/765808/2022. Amsterdam: European Medicines Agency.

[B28] Ferraz M. P. (2025). Wound dressing materials: bridging material science and clinical practice. Appl. Sci. 15, 1725. 10.3390/app15041725

[B29] Ferreira G. d.S. Silva D. J. d. Souza A. G. Yudice E. D. C. Campos I. B. d. Col R. D. (2023). Eco-friendly and effective antimicrobial Melaleuca alternifolia essential oil Pickering emulsions stabilized with cellulose nanofibrils against bacteria and SARS-CoV-2. Int. J. Biol. Macromol. 243, 125228. 10.1016/j.ijbiomac.2023.125228 37290544

[B30] Fierascu R. C. Fierascu I. Baroi A. M. Ortan A. (2021). Selected aspects related to medicinal and aromatic plants as alternative sources of bioactive compounds. Int. J. Mol. Sci. 22, 1521. 10.3390/ijms22041521 33546333 PMC7913593

[B31] Francisconi R. S. Huacho P. M. M. Tonon C. C. Bordini E. A. F. Correia M. F. Sardi J. C. O. (2020). Antibiofilm efficacy of tea tree oil and of its main component terpinen-4-ol against Candida albicans. Braz. Oral Res. 34, e050. 10.1590/1807-3107bor-2020.vol34.0050 32578760

[B32] Gabbai-Armelin P. R. Sales L. S. Ferrisse T. M. De Oliveira A. B. De Oliveira J. R. Giro E. M. A. (2022). A systematic review and meta-analysis of the effect of thymol as an anti-inflammatory and wound healing agent: a review of thymol effect on inflammation and wound healing. Phytother. Res. 36 (9), 3415–3443. 10.1002/ptr.7541 35848908

[B33] Gago C. Serralheiro A. Miguel M. d.G. (2025). Anti-Inflammatory activity of Thymol and thymol-rich essential oils: mechanisms, applications, and recent findings. Molecules 30, 2450. 10.3390/molecules30112450 40509336 PMC12155930

[B34] Gallart-Mateu D. Largo-Arango C. D. Larkman T. Garrigues S. de la Guardia M. (2018). Fast authentication of tea tree oil through spectroscopy. Talanta 189, 404–410. 10.1016/j.talanta.2018.07.023 30086939

[B35] Gan C. Langa E. Valenzuela A. Ballestero D. Pino-Otín M. R. (2023). Synergistic activity of thymol with commercial antibiotics against critical and high WHO priority pathogenic bacteria. Plants 12, 1868. 10.3390/plants12091868 37176927 PMC10180827

[B36] Garapati B. Malaiappan S. Navya P. D. (2023). Evaluation of the degradation, microbial colonization, sustainability of physical and mechanical properties of lycopene coated suture materials-an *in-vitro* Study. J. Popul. Ther. Clin. Pharmacol. 30 (10), E101–E107. 10.47750/jptcp.2023.30.10.015

[B37] Giotopoulou I. Stamatis H. Barkoula N.-M. (2024). Encapsulation of thymol in ethyl cellulose-based microspheres and evaluation of its sustained release for food applications. Polymers 16, 3396. 10.3390/polym16233396 39684141 PMC11644189

[B38] Giráldez-Pérez R. M. Grueso E. M. Carbonero A. Álvarez Márquez J. Gordillo M. Kuliszewska E. (2023). Syn-ergistic antibacterial effects of amoxicillin and gold nanoparticles: a therapeutic option to combat antibiotic resistance. Antibiotics 12, 1275. 10.3390/antibiotics12081275 37627696 PMC10451730

[B39] Hajibonabi A. Yekani M. Sharifi S. Nahad J. S. Dizaj S. M. Memar M. Y. (2023). Antimicrobial activity of nanoformulations of carvacrol and thymol: new trend and applications. OpenNano 13, 100170. 10.1016/j.onano.2023.100170

[B40] Halawa E. M. Fadel M. Al-Rabia M. W. Behairy A. Nouh N. A. Abdo M. (2024). Antibiotic action and resistance: updated review of mechanisms, spread, influencing factors, and alternative approaches for combating resistance. Front. Pharmacol. 14, 1305294. 10.3389/fphar.2023.1305294 38283841 PMC10820715

[B41] Ibrahium S. M. Wahba A. A. Farghali A. A. Abdel-Baki A.-A. S. Mohamed S. A. A. Al-Quraishy S. (2022). Acaricidal activity of tea tree and lemon oil nanoemulsions against Rhipicephalus annulatus. Pathogens 11, 1506. 10.3390/pathogens11121506 36558840 PMC9787657

[B42] International Organization for Standardization (ISO) (2025). Essential Oil of Melaleuca, Terpinen-4-ol Type (Tea Tree Oil). ISO 4730:2025. Geneva: International Organization for Standardization.

[B43] Iseppi R. Mariani M. Condò C. Sabia C. Messi P. (2021). Essential oils: a natural weapon against antibiotic-resistant bacteria responsible for nosocomial infections. Antibiotics 10, 417. 10.3390/antibiotics10040417 33920237 PMC8070240

[B44] Iseppi R. Mariani M. Benvenuti S. Truzzi E. Messi P. (2023). Effects of Melaleuca Alternifolia chell (tea tree) and Eucalyptus globulus labill. Essential oils on antibiotic-resistant bacterial biofilms. Molecules 28, 1671. 10.3390/molecules28041671 36838657 PMC9961662

[B45] Janotto L. d.S. Nazareth T. d.M. Meca G. Luciano F. B. Evangelista A. G. (2023). Exploring the efficacy of antibiotic-essential oil combinations: implications for combating antimicrobial resistance. Bioresour. Technol. Rep. 24, 101679. 10.1016/j.biteb.2023.101679

[B46] Johnson J. B. Thani P. R. Naiker M. (2022). Detection of eucalyptus oil adulteration in Australian tea tree oil using UV–Vis and fluorescence spectroscopy. Talanta Open 6, 100169. 10.1016/j.talo.2022.100169 35988468

[B47] Kairey L. Agnew T. Bowles E. J. Barkla B. J. Wardle J. Lauche R. (2023). Efficacy and safety of Melaleuca alternifolia (tea tree) oil for human health—A systematic review of randomized controlled trials. Front. Pharmacol. 14, 1116077. 10.3389/fphar.2023.1116077 37033604 PMC10080088

[B48] Kamel R. Afifi S. M. Abdou A. M. Esatbeyoglu T. AbouSamra M. M. (2022). Nanolipogel loaded with tea tree oil for the management of burn: GC-MS analysis, *in vitro* and *in vivo* evaluation. Molecules 27, 6143. 10.3390/molecules27196143 36234697 PMC9570711

[B49] Kowalczyk A. Przychodna M. Sopata S. Bodalska A. Fecka I. (2020). Thymol and thyme essential oil—new insights into selected therapeutic applications. Molecules 25, 4125. 10.3390/molecules25184125 32917001 PMC7571078

[B50] Langeveld W. T. Veldhuizen E. J. A. Burt S. A. (2014). Synergy between essential oil components and antibiotics: a review. Crit. Rev. Microbiol. 40 (1), 76–94. 10.3109/1040841X.2013.763219 23445470

[B51] Li X. Shen D. Zang Q. Qiu Y. Yang X. (2021). Chemical components and antimicrobial activities of tea tree hydrosol and their correlation with tea tree oil. Nat. Product. Commun. 16, 1934578X211038390. 10.1177/1934578X211038390

[B52] Liu T. Wang J. Gong X. Wu X. Liu L. Chi F. (2020). Rosemary and Tea Tree essential oils exert antibiofilm activities *in vitro* against Staphylococcus aureus and Escherichia coli. J. Food Prot. 83, 1261–1267. 10.4315/0362-028X.JFP-19-337 32577759

[B53] Lopes A. I. Pintado M. M. Tavaria F. K. (2024). Plant-based films and hydrogels for wound healing. Microorganisms 12, 438. 10.3390/microorganisms12030438 38543489 PMC10972459

[B54] Lupia C. Castagna F. Bava R. Naturale M. D. Zicarelli L. Marrelli M. (2024). Use of essential oils to counteract the phenomena of antimicrobial resistance in livestock species. Antibiotics 13, 163. 10.3390/antibiotics13020163 38391549 PMC10885947

[B55] Maggio F. Rossi C. Serio A. Chaves-Lopez C. Casaccia M. Paparella A. (2025). Anti-biofilm mechanisms of action of essential oils by targeting genes involved in quorum sensing, motility, adhesion, and virulence: a review. Int. J. Food Microbiol. 426, 110874. 10.1016/j.ijfoodmicro.2024.110874 39244811

[B56] Makrygiannis I. H. Nikolaidis A. K. Tilaveridis I. Kouvelas A. D. Lykakis I. Ν. Venetis G. (2025). Coated sutures for use in oral surgery: a comprehensive review. Clin. Oral Invest. 29, 109. 10.1007/s00784-025-06176-w 39903293 PMC11794639

[B57] Malzcak I. Gajda A. (2023). Interactions of naturally occurring compounds with antimicrobials. J. Pharm. Anal. 13 (12), 1452–1470. 10.1016/j.jpha.2023.09.014 38223447 PMC10785267

[B58] Manzanelli F. A. Ravetti S. Brignone S. G. Garro A. G. Martínez S. R. Vallejo M. G. (2023). Enhancing the functional properties of tea tree oil: *in vitro* antimicrobial activity and microencapsulation strategy. Pharmaceutics 15, 2489. 10.3390/pharmaceutics15102489 37896249 PMC10610334

[B59] Maraki S. Mavromanolaki V. E. Kasimati A. Iliaki-Giannakoudaki E. Stafylaki D. (2025). The evolving epidemiology of antimi-crobial resistance of ESKAPE pathogens isolated in the intensive care unit of a Greek university hospital. Diagnostic Microbiol. Infect. Dis. 112, 116804. 10.1016/j.diagmicrobio.2025.116804 40117865

[B60] Marchese A. Orhan I. E. Daglia M. Barbieri R. Di Lorenzo A. Nabavi S. F. (2016). Antibacterial and antifungal activities of thymol: a brief review of the literature. Food Chem. 210, 402–414. 10.1016/j.foodchem.2016.04.111 27211664

[B61] Maria C. Tsolakidou P. Anna K. M. Ilias F. S. Sotiris V. Maria M. (2025). First report of high-risk car-bapenem-resistant K. pneumoniae ST307 clone producing KPC-2, SHV-106, CTX-M-15, and VEB-1 in Greece. Antibiotics 14, 567. 10.3390/antibiotics14060567 40558157 PMC12189958

[B62] Meeran M. F. N. Javed H. Taee H. A. Azimullah S. Ojha S. K. (2017). Pharmacological properties and molecular mechanisms of thymol: prospects for its therapeutic potential and pharmaceutical development. Front. Pharmacol. 8, 380. 10.3389/fphar.2017.00380 28694777 PMC5483461

[B63] Min T. S. Perveen N. Khan N. H. (2020). Comparative purity Study by UV spectrophotometric and fourier-transform infrared spectroscopic (FTIR) techniques for the simultaneous determination of Amoxicillin tri-hydrate capsules. Biomed. J. Sci. and Tech. Res. 31, 24219–24235. 10.26717/BJSTR.2020.31.005104

[B64] Naghavi M. Vollset S. E. Ikuta K. S. Swetschinski L. R. Gray A. P. Wool E. E. (2024). Global burden of bacterial antimicrobial resistance 1990–2021: a systematic analysis with forecasts to 2050. Lancet 404, 1199–1226. 10.1016/s0140-6736(24)01867-1 39299261 PMC11718157

[B65] Najafloo R. Behyari M. Imani R. Nour S. (2020). A mini-review of thymol incorporated materials: applications in antibacterial wound dressing. J. Drug Deliv. Sci. Technol. 60, 101904. 10.1016/j.jddst.2020.101904

[B66] Najjar K. Bridge C. M. (2020). SEM-EDS analysis and characterization of glitter and shimmer cosmetic particles. Forensic Science International 317, 110527. 10.1016/j.forsciint.2020.110527 33065447

[B67] Nascimento T. Gomes D. Simões R. da Graça Miguel M. (2023). Tea tree oil: properties and the therapeutic approach to Acne—A review. Antioxidants 12, 1264. 10.3390/antiox12061264 37371994 PMC10295805

[B68] Nazzaro F. Fratianni F. De Martino L. Coppola R. De Feo V. (2013). Effect of essential oils on pathogenic bacteria. Pharmaceuticals 6 (12), 1451–1474. 10.3390/ph6121451 24287491 PMC3873673

[B69] Nikolic I. Aleksic Sabo V. Gavric D. Knezevic P. (2024). Anti-Staphylococcus aureus activity of Volatile phytochemicals and their combinations with conventional antibiotics against Methicillin-Susceptible *S. aureus* (MSSA) and methicillin-resistant *S. aureus* (MRSA) strains. Antibiotics 13, 1030. 10.3390/antibiotics13111030 39596725 PMC11591321

[B70] Oliveira M. Antunes W. Mota S. Madureira-Carvalho Á. Dinis-Oliveira R. J. Dias da Silva D. (2024). An overview of the recent advances in antimicrobial resistance. Microorganisms 12, 1920. 10.3390/microorganisms12091920 39338594 PMC11434382

[B71] Pannakal S. T. Prasad A. Phadke S. Sanyal A. Butti S. Khodr A. (2025). Acnocure, a synergistic anti-microbial and anti-inflammatory combination of Thymol and curcuma turmerones, formulation and time-kill studies against C. acnes. Cosmetics 12, 37. 10.3390/cosmetics12020037

[B72] Parolin G. A. Vital V. G. de Vasconcellos S. P. Lago J. H. G. Péres L. O. (2024). Thymol as starting material for the development of a biobased material with enhanced antimicrobial activity: synthesis, characterization, and potential application. Molecules 29, 1010. 10.3390/molecules29051010 38474522 PMC10933892

[B73] Pezantes-Orellana C. Bermúdez F. G. De la Cruz C. M. Montalvo J. L. Orellana-Manzano A. (2024). Essential oils: a systematic review on revolutionizing health, nutrition, and omics for optimal well-being. Front. Med. 11, 1337785. 10.3389/fmed.2024.1337785 PMC10905622 38435393

[B74] Pinto L. Baruzzi F. Terzano R. Busto F. Marzulli A. Magno C. (2024). Analytical and antimicrobial characterization of Zn-Modified clays embedding thymol or carvacrol. Mol. Basel, Switz. 29 (15), 3607. 10.3390/molecules29153607 PMC11313700 39125013

[B75] Raikwar G. Kumar D. Mohan S. Dahiya P. (2024). Synergistic potential of essential oils with antibiotics for antimicrobial resistance with emphasis on mechanism of action: a review. Biocatal. Agric. Biotechnol. 61, 103384. 10.1016/j.bcab.2024.103384

[B76] Rawat P. Singh R. N. Ranjan A. Ahmad S. Saxena R. (2017). Antimycobacterial, antimicrobial activity, experimental (FT-IR, FT-Raman, NMR, UV-Vis, DSC) and DFT (transition state, chemical reactivity, NBO, NLO) studies on pyrrole-isonicotinyl hydrazine. Spectrochimica Acta. Part A, Mol. Biomolecular Spectroscopy 179, 1–10. 10.1016/j.saa.2017.02.021 28213139

[B77] Romanescu M. Oprean C. Lombrea A. Badescu B. Teodor A. Constantin G. D. (2023). Current State of knowledge regarding WHO high priority Pathogens—Resistance mechanisms and proposed solutions through candidates such as essential oils: a systematic review. Int. J. Mol. Sci. 24, 9727. 10.3390/ijms24119727 37298678 PMC10253476

[B78] Rosa V. Silikas N. Yu B. Dubey N. Sriram G. Zinelis S. (2024). Guidance on the assessment of biocompatibility of biomaterials: fundamentals and testing considerations. Dent. Materials Official Publication Acad. Dent. Mater. 40 (11), 1773–1785. 10.1016/j.dental.2024.07.020 39129079

[B79] Ruiz H. K. Ruiz M. Cabañas A. Calvo L. (2024). Inactivation of Staphylococcus epidermidis in a cotton gauze with supercritical CO2 modified with essential oils. Processes 12, 2158. 10.3390/pr12102158

[B80] Sakalauskienė G. V. Malcienė L. Stankevičius E. Radzevičienė A. (2025). Unseen enemy: mechanisms of multidrug antimicrobial resistance in gram-negative ESKAPE pathogens. Antibiotics 14, 63. 10.3390/antibiotics14010063 39858349 PMC11762671

[B81] Sánchez García E. Torres-Alvarez C. Morales Sosa E. G. Pimentel-González M. Villarreal Treviño L. Amaya Guerra C. A. (2024). Essential oil of fractionated oregano as motility inhibitor of bacteria associated with urinary tract infections. Antibiotics 13, 665. 10.3390/antibiotics13070665 39061347 PMC11273670

[B82] Scientific Committee on Consumer Safety (SCCS) (2025). Scientific Opinion on Tea Tree Oil (CAS/EC No. 68647-73-4/285-377-1) Used in Cosmetic Products. SCCS/1681/25. Brussels: European Commission.

[B83] Sharma K. Munjal M. Sharma R. K. Sharma M. (2023). Thymol encapsulated chitosan-Aloe vera films for antimicrobial infection. Int. J. Biol. Macromol. 235, 123897. 10.1016/j.ijbiomac.2023.123897 36870638

[B84] Simbu S. Orchard A. Vuuren S. v. (2024). Essential oil compounds in combination with conventional antibiotics for dermatology. Molecules 29, 1225. 10.3390/molecules29061225 38542862 PMC10974782

[B85] Sobieszczański J. Mertowski S. Sarna-Boś K. Stachurski P. Grywalska E. Chałas R. (2023). Root canal infection and its impact on the oral cavity microenvironment in the context of immune System disorders in selected diseases: a narrative review. J. Clin. Med. 12, 4102. 10.3390/jcm12124102 37373794 PMC10298853

[B86] Sodhi K. K. Kumar M. Singh D. K. (2021). Insight into the amoxicillin resistance, ecotoxicity, and remediation strategies. J. Water Process Eng. 39, 101858. 10.1016/j.jwpe.2020.101858

[B87] Soulaimani B. (2025). Comprehensive review of the combined antimicrobial activity of essential oil mixtures and synergism with conventional antimicrobials. Nat. Prod. Commun. 20, 1934578X251328241. 10.1177/1934578x251328241

[B88] Stefănescu R. Laczkó-Zöld E. Ciurea C. Tero-Vescan A. Ősz B. Vancea S. (2025). GC-MS profiling and antimicrobial activity of eight essential oils against opportunistic pathogens with biofilm-forming potential. Int. J. Mol. Sci. 26, 10928. 10.3390/ijms262210928 41303410 PMC12652525

[B89] Stohr J. J. J. M. Kluytmans-van den Bergh M. F. Q. Verhulst C. J. M. M. Rossen J. W. A. Kluytmans J. A. J. W. (2020). Development of amoxicillin resistance in Escherichia coli after exposure to remnants of a non-related phagemid-containing E. coli: an exploratory study. Antimicrob. Resist. Infect. Control 9, 48. 10.1186/s13756-020-00708-7 32178740 PMC7077161

[B90] Szabelski J. Karpiński R. (2024). Short-Term hydrolytic degradation of mechanical properties of absorbable surgical sutures: a comparative Study. J. Funct. Biomater. 15, 273. 10.3390/jfb15090273 39330248 PMC11432777

[B91] Taheri-Araghi S. (2024). Synergistic action of antimicrobial peptides and antibiotics: current understanding and future directions. Front. Microbiol. 14, 1390765. 10.3389/fmicb.2024.1390765 PMC11322369 39144233

[B92] Tisserand R. Young R. , (2014). Essential OIL SAFETY—A guide for health care professionals| Robert Tisserand and Rodney Young, Essential Oil Safety—A Guide for Health Care Professionals. 2nd Edn. Edinburgh: Churchill Livingstone. 10.1016/B978-0-443-06241-4.09991-4

[B93] Touati A. Mairi A. Ibrahim N. A. Idres T. (2025). Essential oils for biofilm control: mechanisms, synergies, and translational challenges in the era of antimicrobial resistance. Antibiotics 14, 503. 10.3390/antibiotics14050503 40426569 PMC12108346

[B94] Vassiliou E. Awoleye O. Davis A. Mishra S. (2023). Anti-Inflammatory and antimicrobial properties of thyme oil and its main constituents. Int. J. Mol. Sci. 24, 6936. 10.3390/ijms24086936 37108100 PMC10138399

[B95] Wang L. Zhang Y. Liu L. Huang F. Dong B. (2021). Effects of three-layer encapsulated tea tree oil on growth performance, antioxidant capacity, and intestinal microbiota of weaned pigs. Front. Vet. Sci. 8, 789225. 10.3389/fvets.2021.789225 34926648 PMC8674471

[B96] Yasin M. Younis A. Javed T. Akram A. Ahsan M. Shabbir R. (2021). River tea tree oil: composition, antimicrobial and antioxidant activities, and potential applications in agriculture. Plants 10, 2105. 10.3390/plants10102105 34685914 PMC8540646

[B97] Yu Z. Tang J. Khare T. Kumar V. (2020). The alarming antimicrobial resistance in ESKAPEE pathogens: can essential oils come to the rescue? Filoterapia 140, 104433. 10.1016/j.fitote.2019.104433 31760066

[B98] Yuan G. Zhou Q. Wang S. Sun H. Wang Y. (2025). Effect of molecular weight of Chitosan on tea tree essential oil-loaded nanoparticles: formation, characteristics, and application in preservation of mini-cucumbers. Food Biophys. 20, 30. 10.1007/s11483-024-09923-w

[B99] Zamani Z. Alipour D. Moghimi H. R. Montazavi S. A. R. Saffary M. (2015). Development and evaluation of Thymol microparticles using cellulose derivatives as controlled release dosage form. Iran. J. Pharm. Res. 14 (4), 1031–1040. 26664369 PMC4673930

[B100] Zhou W. Wang Z. Mo H. Zhao Y. Li H. Zhang H. (2019). Thymol mediates bactericidal activity against Staphylococcus aureus by targeting an Aldo–Keto reductase and consequent depletion of NADPH. J. Agric. Food Chem. 67 (30), 8382–8392. 10.1021/acs.jafc.9b03517 31271032

[B101] Zhu L. Liu Y. Liu J. Qiu X. Lin L. (2024). Preparation and characterization of tea tree essential oil microcapsule-coated packaging paper with bacteriostatic effect. Food Chem. X 23, 101510. 10.1016/j.fochx.2024.101510 38947341 PMC11214406

