# Supplementary material for: Sustainable thymol-based formulations with amoxicillin and Melaleuca alternifolia essential oil
Source: Front Chem. 2026 Aug 10;14:1898133. doi: 10.3389/fchem.2026.1898133 (PMC13500663; doi:10.3389/fchem.2026.1898133)
Supplement: Supplementary file 1 [file Supplementaryfile1.docx]

TT2A

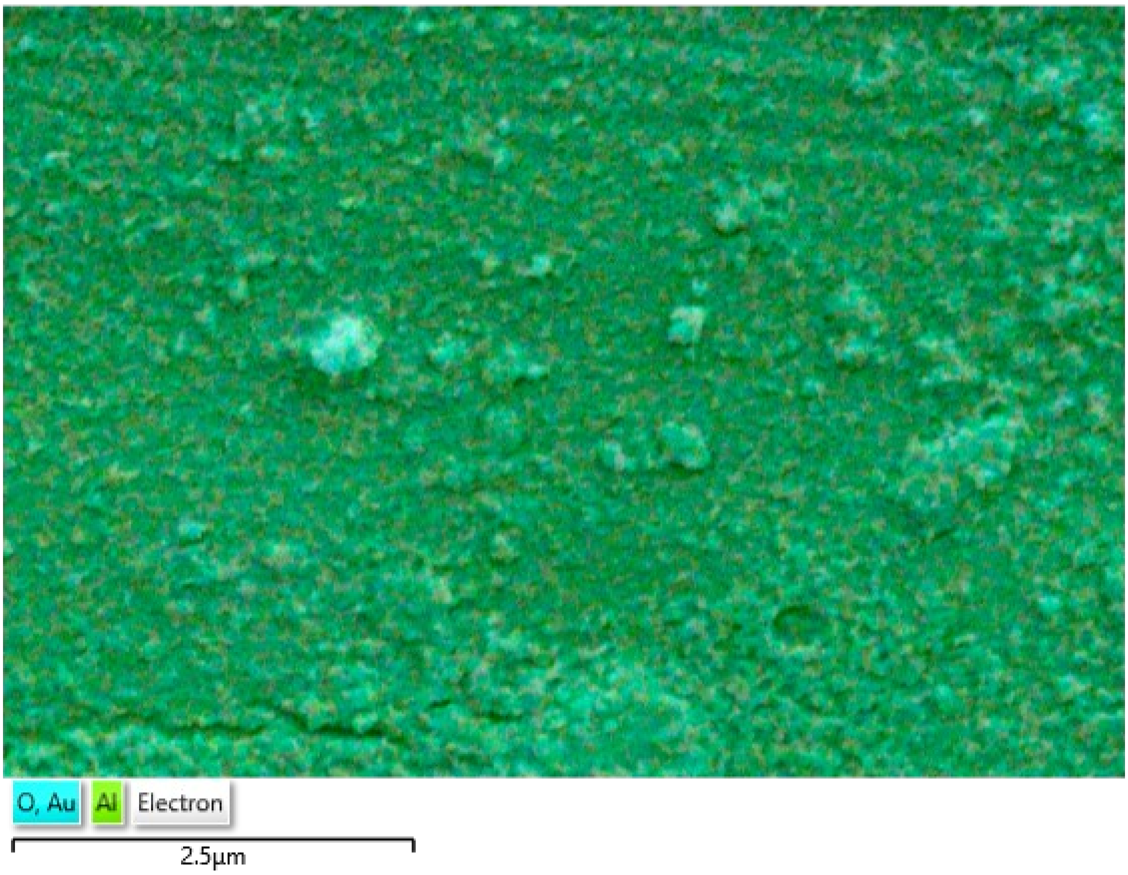

Al Kα1

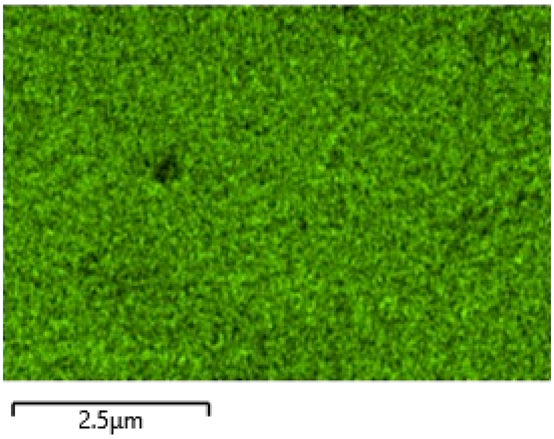

Au Mα1

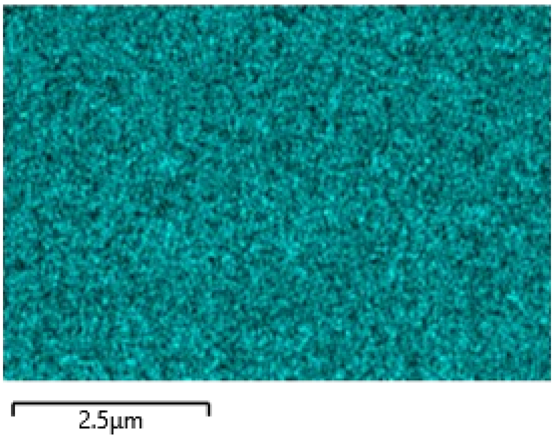

Cu Lα1,2

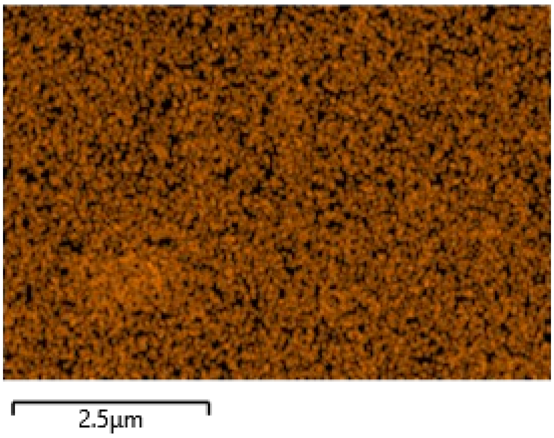

C Kα1,2

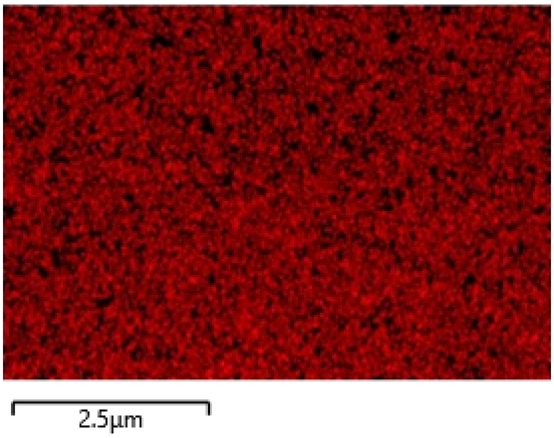

O Kα1

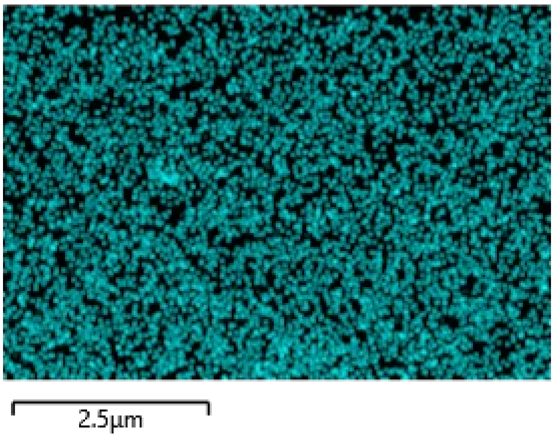

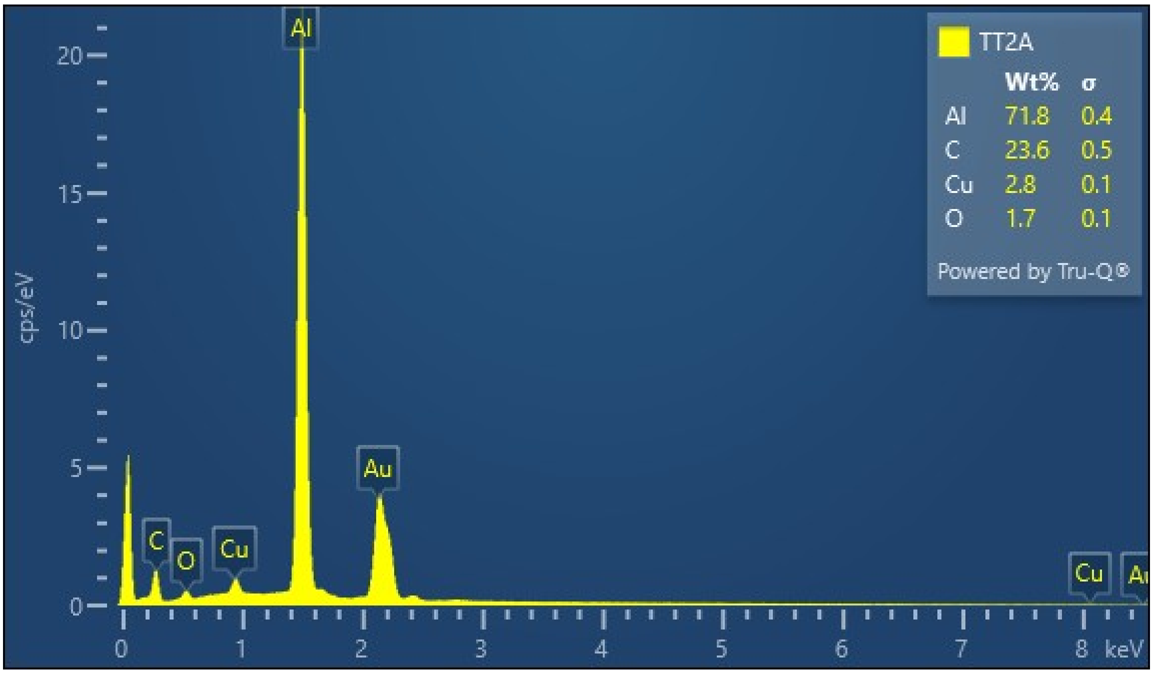
TT2A
Wt% σ
Al 71.8 0.4
C 23.6 0.5
Cu 2.8 0.1
O 1.7 0.1
Powered by Tru-Q®
cps/eV
20
15
10
5
0
Al
Au
C
O
Cu
Cu
0
1
2
3
4
5
6
7
8 keV
