# Supplementary material for: Sustainable thymol-based formulations with amoxicillin and Melaleuca alternifolia essential oil
Source: Front Chem. 2026 Aug 10;14:1898133. doi: 10.3389/fchem.2026.1898133 (PMC13500663; doi:10.3389/fchem.2026.1898133)
Supplement: Supplementary file 4 [file Supplementaryfile4.docx]

Electron Image 12

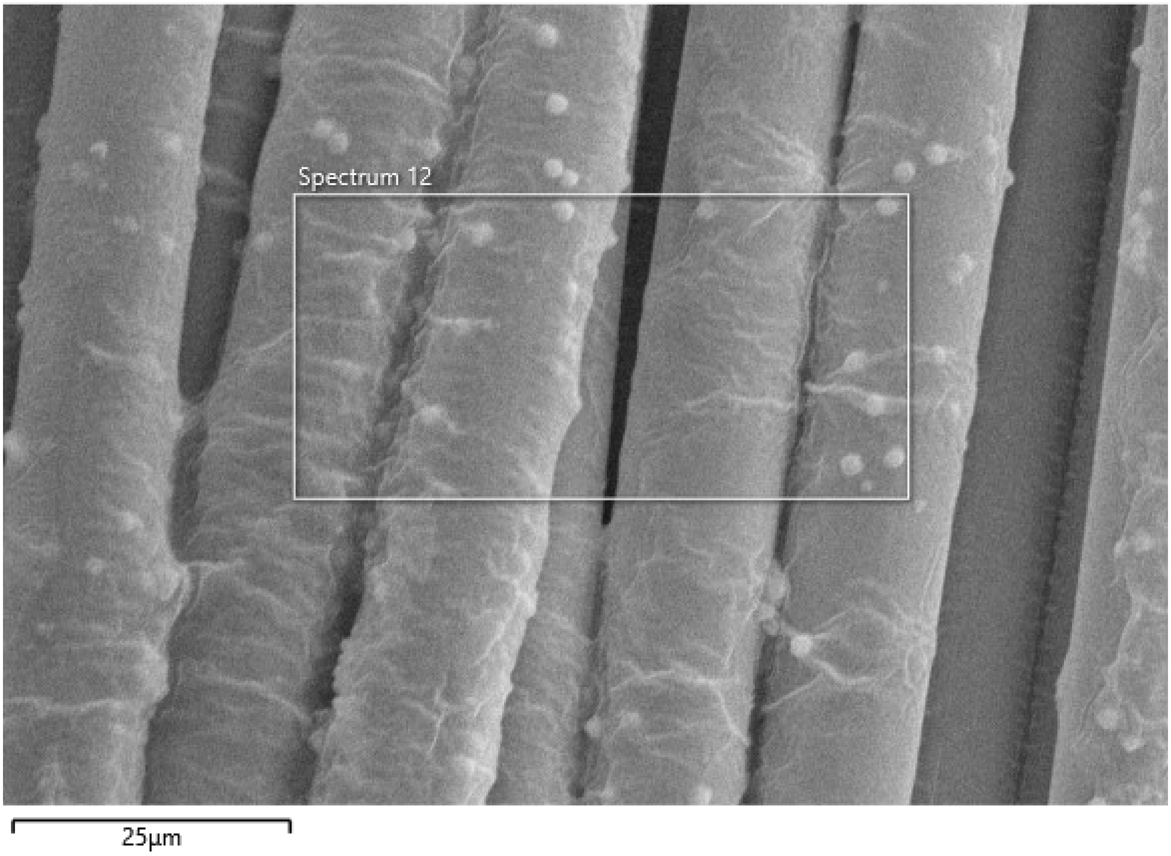

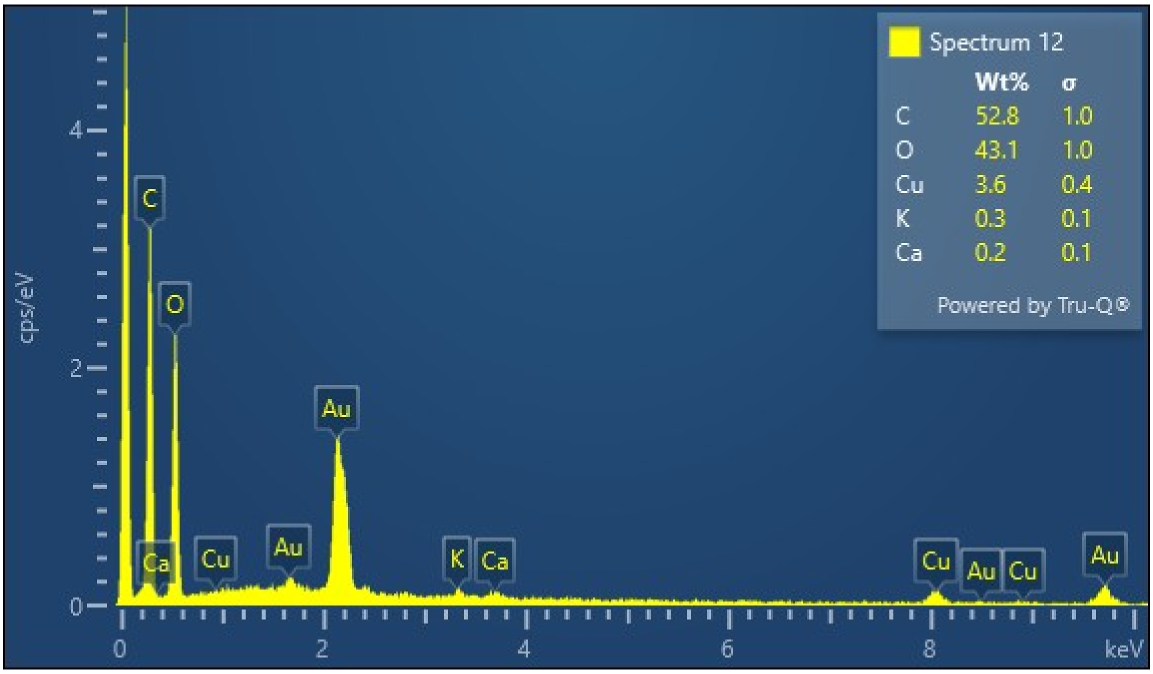

OXFORD INSTRUMENTS

The Business of Science®
